# Boron Difluoride Formazanate Dye With Donor Planarization Engineering for 1060 nm Laser Activated Photothermal Theranostics

**DOI:** 10.1002/advs.202506226

**Published:** 2025-07-06

**Authors:** Kang Xu, Mengchen Luo, Weili Wang, Tian Zhang, Yang Chen, Jinjun Shao, Peng Chen, Xiaochen Dong, Yu Cai

**Affiliations:** ^1^ State Key Laboratory of Flexible Electronics (LoFE) & Institute of Advanced Materials (IAM) School of Flexible Electronics (Future Technologies) Nanjing Tech University Nanjing 211816 China; ^2^ School of Chemistry Chemical Engineering and Biotechnology Institute for Digital Molecular Analytics and Science Lee Kong Chian School of Medicine Nanyang Technological University 62 Nanyang Drive Singapore 637459 Singapore; ^3^ School of Chemistry & Materials Science Jiangsu Normal University Xuzhou 221116 China; ^4^ Center for Rehabilitation Medicine Rehabilitation & Sports Medicine Research Institute of Zhejiang Province Department of Rehabilitation Medicine Cancer Center Zhejiang Provincial People's Hospital (Affiliated People's Hospital) Hangzhou Medical College Hangzhou 310014 China

**Keywords:** donor engineering, NIR‐II, photoacoustic imaging, photothermal therapy

## Abstract

Phototheranostics within the second near‐infrared (NIR‐II, 1000–1700 nm) window offer considerable advantages compared to those operating in the visible and first near‐infrared region (NIR‐I, 700–900 nm). Herein, a donor‐acceptor‐donor (D‐A‐D) structured NIR‐II absorbing Boron Difluoride Formazanate (BDF) dye FBDFDPA is developed through a donor engineering strategy for 1060 nm laser‐excited photoacoustic imaging (PAI)‐guided photothermal therapy (PTT). By replacing the donor unit from alkoxy‐substituted triphenylamine with alkoxy‐substituted diphenylamine, the planarity of the molecular conjugated skeleton is enhanced, thus promoting the intramolecular charge transfer (ICT) effect, leading to a 138 nm redshift in absorption and extending it into the NIR‐II region. FBDFDPA NPs show excellent molar extinction coefficient (ε = 7.66 × 10^3^ M^−1^ cm^−1^) at 1060 nm and significant photothermal conversion efficiency (*η* = 54.4%), enabling effective NIR‐II PTT for tumor ablation under the guidance of NIR‐II PAI. This work establishes a donor planarization engineering strategy for designing NIR‐II absorbing dyes, offering a promising avenue for advanced cancer photothermal theranostics.

## Graphical abstract

The donor engineering strategy enhances the planarity of the π‐conjugated molecular framework, thereby promoting stronger intramolecular charge transfer (ICT). This structural refinement in FBDFDPA leads to a pronounced bathochromic shift in absorption from the NIR‐I to the NIR‐II region, enabling effective photothermal therapy (PTT) for tumors within the NIR‐II window.

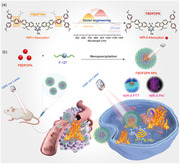

## 1 Introduction

Cancer remains a leading global health burden, despite significant advancements in diagnostic and therapeutic strategies.^[^ ^1^ ^]^ Phototherapy, as an innovative cancer treatment approach, has attracted significant interest owing to its potential efficacy and unique advantages. Unlike conventional therapeutic approaches, phototherapy enables real‐time diagnosis and simultaneous in situ treatment during laser irradiation. Among these techniques, photothermal therapy (PTT) leverages external light to generate localized heat, effectively inducing tumor ablation in a non‐invasive and light‐controlled manner. This approach has shown great promise as an emerging, minimally invasive cancer treatment in the medical community.^[^ ^2^ ^]^ In recent years, various photothermal agents, mainly including metal nanoparticles,^[^ ^3^ ^]^ carbon nanotubes,^[^ ^4^ ^]^ transition metal dichalcogenides (TMDCs),^[^ ^5^ ^]^ π‐conjugated materials,^[^ ^6^ ^]^ and Mxenes,^[^ ^7^ ^]^ have been extensively explored for PTT. Notably, organic photothermal agents, such as near‐infrared (NIR) dyes and π‐conjugated semiconducting polymers, have drawn significant interest due to their intrinsic biocompatibility, as well as their highly tunable chemical structures and photophysical properties^[^ ^8^ ^]^.

The close relationship between photoacoustic imaging (PAI) and PTT has led to their frequent combined use for PAI‐guided PTT. This phototheranostic approach relies on photothermal agents capable of effectively transforming light energy into thermal energy, thereby inducing cell death through PTT.^[^ ^9^ ^]^ Simultaneously, as‐generated heat can promote the expansion of adjacent tissues, resulting in the production of ultrasound waves that can be transformed into imaging signals for PAI.^[^ ^10^ ^]^ In comparison to the first near‐infrared region (NIR‐I, 700–900 nm), NIR‐II (1000–1700 nm) photoirradiation exhibits enhanced tissue penetration depth and allows for higher permissible exposure thresholds, which significantly reduces the risk of phototoxicity while enhancing the precision of photothermal therapy.^[^ ^11^ ^]^ The exploration of organic molecules with NIR‐II absorption properties commonly employs molecular engineering strategies of constructing the donor‐acceptor‐donor (D‐A‐D) architectures, extending the π‐conjugation, and fusing rings to create a sizable π‐conjugated framework^[^ ^12^ ^]^.

Recently, BF_2_ complexes have attracted considerable research attention and have been widely used as optoelectronic materials and phototheranostic agents.^[^ ^13^ ^]^ Boron dipyrromethene (BODIPY), one of the most popular BF_2_ complexes, exhibits intriguing photophysical and electrochemical properties, owing to the boron‐nitrogen coordination bond (B←N) generated from the chelation between dipyrromethene and Boron trifluoride‐diethyl etherate (BF_3_·OEt_2_).^[^ ^14^ ^]^ Similarly, the Boron Difluoride Formazanate (BDF) dyes, with two B←N bonds embedded within the six‐membered ring, have been employed as a strong electron acceptor to construct NIR molecules for electroluminescence and biophotonics.^[^ ^15^ ^]^ Notably, Gilroy's group has made pioneering contributions to the synthesis and functional exploration of BDF dyes, establishing a comprehensive framework to take advantage of their unique optoelectronic and electrochemical properties in advanced material applications^[^ ^16^ ^]^.

**Scheme 1:**
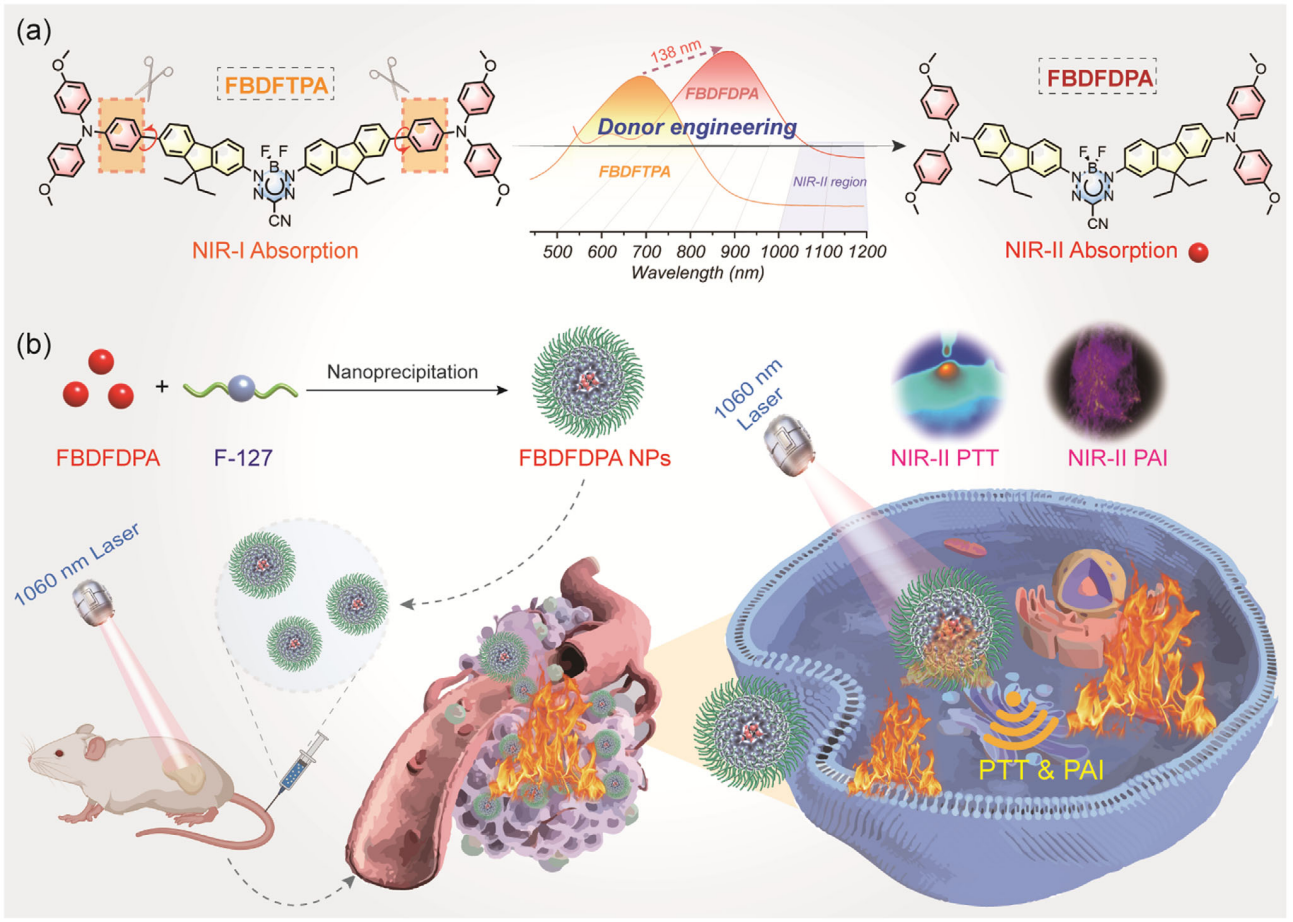
Schematic illustration of the donor engineering strategy and its phototheranostic application on mice triggered by 1060 nm laser.

Herein, we developed a D‐A‐D structured BDF dye FBDFDPA with NIR‐II absorption for NIR‐II PAI‐guided tumor PTT utilizing the donor engineering strategy (**Scheme** **1**). Through changing the donor unit from 4,4′‐dimethoxytriphenylamine to 4,4′‐dimethoxydiphenylamine to enhance the planarity of the molecular conjugated skeleton, thus facilitating the intramolecular charge transfer (ICT), the absorption band of FBDFDPA redshifted from the NIR‐I region to the NIR‐II region. Through using amphiphilic polymer F‐127 to prepare the water‐dispersible nanoparticles (NPs), FBDFDPA NPs exhibited excellent molar extinction coefficient at 1060 nm (ε = 7.66 × 10^3^ M^−1^ cm^−1^) and significant photothermal conversion efficiency (PCE, *η* = 54.4%) under 1060 nm photoirradiation, enabling efficient photothermal tumor ablation guided by NIR‐II PAI. This research provides a general guideline for the molecular design of NIR‐II absorption dyes for cancer phototheranostics.

## 2 Results and Discussion

### 2.1 Design and Characterization of FBDFDPA and FBDFTPA

**Figure 1:**
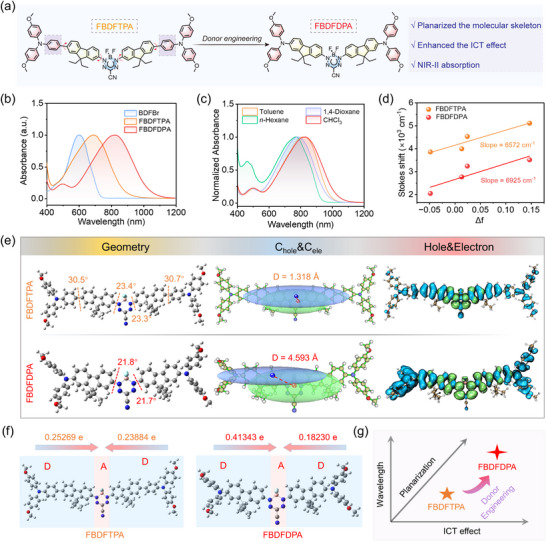
a) The NIR‐II dye FBDFDPA design strategy. b) Normalized absorption of BDFBr, FBDFTPA, and FBDFDPA in DCM. c) Normalized absorption of FBDFDPA in CHCl_3_, 1,4‐Dioxane, Toluene, and *n*‐Hexane. d) Correlation of the Δ*f* of the solvents with the Stokes shift. e) Optimized geometries, C_hole_&C_ele_, and Hole&Electron Graphs of FBDFTPA and FBDFDPA. f) Diagram of the amounts of charge transfer between fragments of FBDFTPA and FBDFDPA upon vertical excitation. g) Property comparison between FBDFDPA and FBDFTPA.

The D‐A‐D structures usually exhibit a pronounced ICT effect, resulting in a lower electronic band gap and longer absorption and emission bands.^[^ ^17^ ^]^ In this study, two D‐A‐D structured dyes, FBDFDPA and FBDFTPA, were synthesized through Pd‐catalyzed Buchwald‐Hartwig cross‐coupling and Suzuki cross‐coupling, respectively, wherein the BDF unit was employed as the electron acceptor and alkoxy‐substituted arylamine was the electron donor (**Figure** **1**a; Figure S1, Supporting Information). The molecular structures of FBDFTPA, FBDFDPA, and their synthetic intermediates were unambiguously characterized by comprehensive spectroscopic analyses, including ^1^H NMR, ^13^C NMR, and matrix‐assisted laser desorption/ionization time‐of‐flight (MALDI‐TOF) mass spectrometry, as detailed in the Supporting Information.

Initially, the photophysical characteristics of FBDFDPA and FBDFTPA were investigated. The UV‐visible‐NIR absorption spectra of FBDFTPA and FBDFDPA in dichloromethane (DCM) are shown in Figure 1b. After introducing arylamine donors at the periphery sites of the BDF core (BDFBr) to construct the D‐A‐D skeleton, the maximum absorption peaks of FBDFTPA and FBDFDPA demonstrated a pronounced bathochromic shift. The maximum absorption peak of FBDFDPA was located at 832 nm, with the tail extending beyond 1100 nm, while the absorption peak of FBDFTPA was at 694 nm, exhibiting a redshift of 232 and 94 nm, respectively, in contrast to the absorption peak of BDFBr. Notably, the replacement of the 4,4′‐dimethoxy‐triphenylamine donor unit with a 4,4′‐dimethoxy‐diphenylamine donor unit in the design of FBDFDPA significantly enhanced ICT efficiency, effectively shifting the absorption band into the NIR‐II region.

Subsequently, the photophysical properties of FBDFDPA and FBDFTPA were studied in organic solvents with varying polarities. It was found that FBDFDPA exhibited a red shift in absorption relative to FBDFTPA across these solvents (Figure 1c; Figure S2, Supporting Information). The Lippert‐Mataga equation elucidates the correlation between the Stokes shift (Δ*ν* = ν_abs_ − ν_em_) and the solvent polarity parameter (Δ*f*). In the Δν∼Δ*f* plot, FBDFDPA demonstrated a steeper slope (6925 cm^−1^) than that of FBDFTPA (6572 cm^−1^), suggesting a more pronounced ICT effect in FBDFDPA (Figure 1d).^[^ ^18^ ^]^ Further, the calculated dipole moment changes (Δµg_e_) between ground and excited states, specifically 17.6 D for FBDFTPA and 18.1 D for FBDFDPA, quantitatively support this observation.

To gain a deeper understanding of the differences in photophysical properties between FBDFTPA and FBDFDPA, comprehensive theoretical investigations were conducted using density functional theory (DFT) with the Gaussian 09 program at the B3LYP/def2‐SVP level. In FBDFDPA, the N‐atom of the diphenylamine (DPA) donor was directly connected to the fluorene unit, while in FBDFTPA, the N‐atom of the Triphenylamine (TPA) donor was connected to the fluorene unit through an additional phenyl ring, resulting in relatively large dihedral angles (30.5° and 30.7°) between the phenyl ring and the fluorene unit (Figure 1e). The non‐planar “propeller” conformation of TPA in FBDFTPA disrupted effective π‐conjugation between the donor and acceptor (D‐A) units, favoring localized excitation (LE) states with limited charge separation efficiency.^[^ ^19^ ^]^ In contrast, the DPA‐containing FBDFDPA exhibited a highly coplanar π‐skeleton, eliminating steric distortion. This rigidity of FBDFDPA favored a delocalized charge transfer (CT) state, effectively reducing the excitation energy (Figure 1g; Figures S3, S4 and Table S2, Supporting Information).

Through donor engineering, a pronounced 138 nm red‐shift in the absorption band was observed from FBDFTPA to FBDFDPA. Then, the electronic excitation characteristics were analyzed by using Multiwfn and VMD.^[^ ^20^ ^]^ Remarkably, the S_0_→S_1_ transition with the highest oscillator strength (*f* = 1.3521) of FBDFDPA corresponds to the intensive absorption peak at 832 nm, while the S_0_→S_3_ transition with the highest oscillator strength (*f* = 1.1362) of FBDFTPA corresponds to the absorption peak at 694 nm (Figures S3, S4, Supporting Information). Notably, the distance between the centroid of the hole and the electron of FBDFDPA (D = 4.593 Å) was much larger than that of FBDFTPA (D = 1.318 Å), demonstrating extended spatial charge separation (Figure 1e). This extended charge separation effectively reduced the energy gap and excitation energy, leading to the red‐shifted absorption peak of FBDFDPA (Figure 1e; Figures S5, S6, Supporting Information). Furthermore, FBDFDPA demonstrated superior hole delocalization indices (HDI = 5.66) and electron delocalization indices (EDI = 9.32), along with a lower electron‐hole overlap (S_HE_ = 0.429 vs 0.624 for FBDFTPA), collectively indicating the enhanced charge transport and separation properties (Figures S5, S6 and Table S2, Supporting Information).^[^ ^21^ ^]^ Crucially, FBDFDPA exhibited larger charge‐transfer amounts (0.59573 e) compared to FBDFTPA (0.49153 e), reflecting a stronger ICT effect for FBDFDP (Figure 1f; Figure S7, Supporting Information).^[^ ^22^ ^]^ These synergistic effects not only amplified the ICT effect but also broadened the red‐shifted absorption band of FBDFDPA.

### 2.2 Preparation and Characterization of FBDFDPA NPs

**Figure 2:**
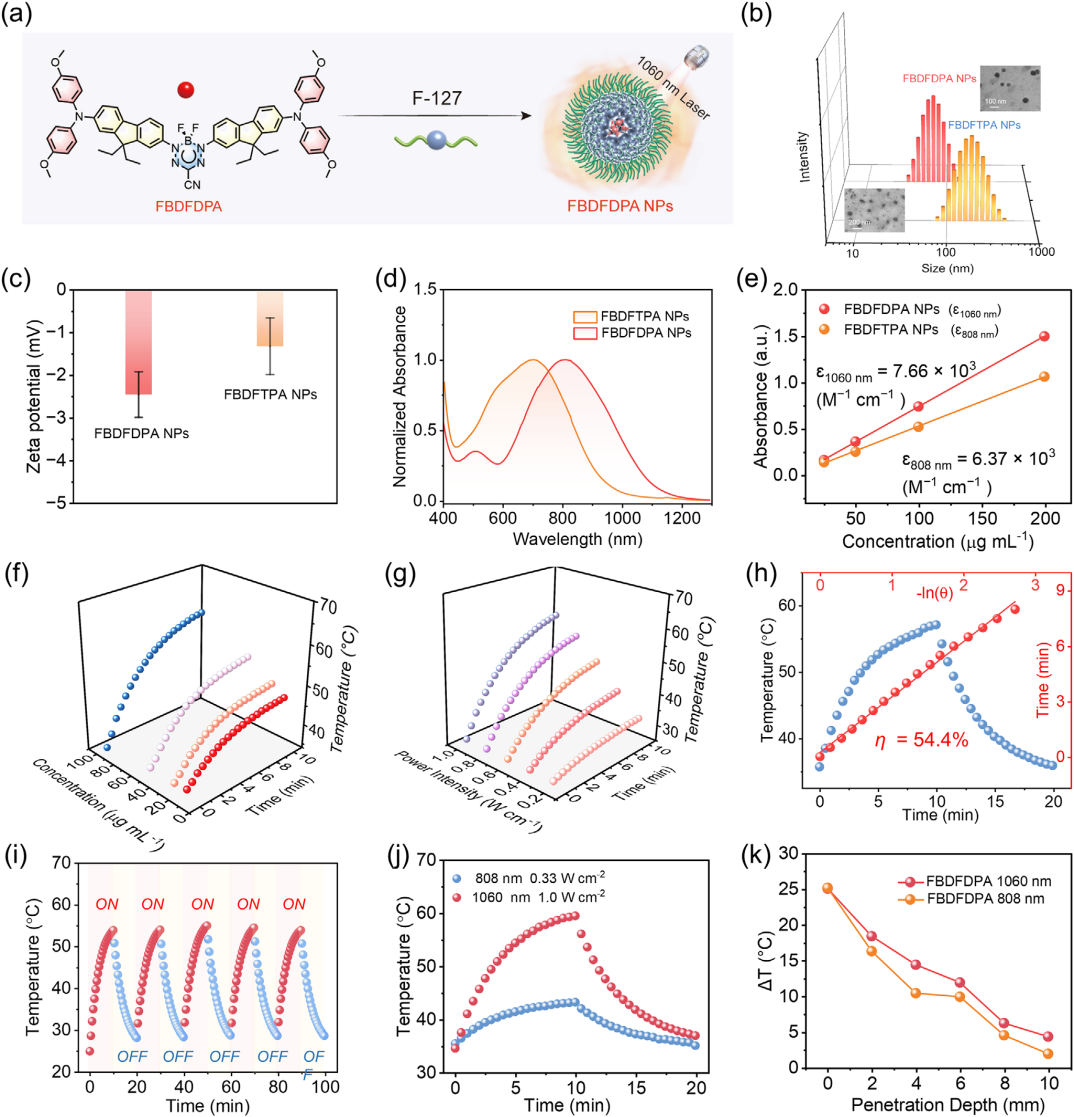
a) The preparation of the FBDFDPA NPs. b) Hydrodynamic diameters and TEM images (inset) of FBDFDPA NPs and FBDFTPA NPs. c) Zeta potential of FBDFDPA NPs and FBDFTPA NPs. d) Normalized absorption spectra of FBDFDPA NPs and FBDFTPA NPs. e) The molar extinction coefficient of FBDFDPA NPs and FBDFTPA NPs. f) Changes of temperature in different concentrations of FBDFDPA NPs and g) different laser power of FBDFDPA NPs (100 µg mL^−1^). h) The PCE calculation of FBDFDPA NPs. i) The photothermal stability assessment of FBDFDPA NPs (100 µg mL^−1^). j) The thermal variations of FBDFDPA NPs subjected to laser irradiation at 808 nm (0.33 W cm^−2^) and 1060 nm (1.0 W cm^−2^). k) Comparison of temperature increment of FBDFDPA NPs covering different thicknesses of chicken breast tissue under 808 and 1060 laser irradiation.

To enhance water solubility, the amphiphilic Pluronic F‐127 was utilized as an encapsulation matrix for these hydrophobic dyes through a nanoprecipitation method (**Figure** **2**a). Dynamic light scattering (DLS) analysis revealed that FBDFDPA NPs and FBDFTPA NPs exhibited well‐defined hydrodynamic diameters of 61.7 and 167.5 nm, respectively, demonstrating their favorable dispersibility in aqueous media (Figure 2b). Morphological characterization through transmission electron microscopy (TEM) further confirmed that both FBDFDPA NPs and FBDFTPA NPs exhibited monodisperse spherical architectures with uniform size distributions. Moreover, the zeta potentials of FBDFDPA NPs and FBDFTPA NPs were recorded at ‐2.47 and ‐1.33 mV, respectively (Figure 2c). The appropriate dimensions and surface charge of FBDFDPA NPs and FBDFTPA NPs facilitated the effective circulation within the bloodstream and enhanced their accumulation in tumor sites^[^ ^23^ ^]^.

Notably, FBDFDPA NPs exhibited a broader absorption spectrum within the NIR‐II window range of 1000–1200 nm, compared to FBDFTPA NP (Figure 2d). Furthermore, the molar extinction coefficient of FBDFDPA NPs at 1060 nm was determined to be 7.66 × 10^3^ M^−1^ cm^−1^, whereas that of FBDFTPA NPs at 808 nm was measured at 6.37 × 10^3^ M^−1^ cm^−1^ (Figure 2e; Figure S8, Supporting Information). These findings demonstrate that FBDFDPA NPs could be a prominent candidate for NIR‐II PTT.

The stability of FBDFDPA NPs under laser irradiation was subsequently assessed. In comparison to the clinical dye indocyanine green (ICG), FBDFDPA NPs demonstrated enhanced photostability. Following 18‐min exposure to the 1060 nm laser irradiation (1.0 W cm^−2^), the absorbance of FBDFDPA NPs remained relatively unchanged, whereas the characteristic absorption peak of ICG exhibited a rapid decline (Figure S9, Supporting Information).

Since FBDFTPA NPs and FBDFDPA NPs exhibited intense NIR absorption, their photothermal performance was evaluated by recording the temperature changes. Upon irradiation with NIR‐II (1060 nm) and NIR‐I (808 nm) laser, FBDFDPA NPs and FBDFTPA NPs exhibited characteristic photothermal effects that were dependent on both concentration and laser power density (Figure 2f,g; Figure S10, Supporting Information). After 10 min of laser irradiation, the temperatures of FBDFDPA NPs and FBDFTPA NPs (100 µg mL^−1^) reached 59.5 and 48.9 °C, respectively. It is worth mentioning that even at a minimal concentration of 12.5 µg mL^−1^, the temperature of FBDFDPA NPs could increase to 47.0 °C, reaching the temperature threshold for tumor cell ablation. Then, according to the cooling phase of the photothermal curves revealed that FBDFDPA NPs achieved a remarkable PCE of 54.4% under NIR‐II photoirradiation (1060 nm, 1.0 W cm^−^^2^) while maintaining a high efficiency of 49.4% under NIR‐I photoirradiation (808 nm, 1.0 W cm^−^^2^).^[^ ^24^ ^]^ In contrast, FBDFTPA NPs exhibited a comparable PCE of 47.3% under 808 nm photoirradiation (Figure 2h; Figure S10, Supporting Information).

Subsequently, the photostability of FBDFDPA NPs and FBDFTPA NPs was evaluated by monitoring temperature variations during a series of alternating heating‐cooling cycles. Following five cycles, there was a minimal change observed in the photothermal performance of FBDFDPA NPs and FBDFTPA NPs (Figure 2i; Figure S10, Supporting Information). Given that the safe maximum permissible exposure (MPE) density of the 808 and 1060 nm lasers is 0.33 and 1.0 W cm^−2^, respectively, the photothermal efficacy of FBDFDPA NPs was assessed at the safe laser power density levels. Under conditions of safe MPE intensity, the temperature increase resulting from 10‐min 1060 nm laser irradiation was significantly greater than that observed with the 808 nm laser (Figure 2j). These results underscored the potential of FBDFDPA NPs for NIR‐II PTT.

After that, the penetration depth capabilities of 808 and 1060 nm lasers were investigated. To simulate tissue penetration characteristics, the laser detection system was systematically evaluated through chicken breast tissue phantoms of varying thicknesses (0‐10 mm) under controlled irradiation conditions (0.2–1.0 W cm^−2^) at NIR‐I (808 nm) and NIR‐II (1060 nm) wavelengths (Figure S11, Supporting Information).^[^ ^25^ ^]^ The transmittance of the 1060 nm laser demonstrated a higher efficacy compared to the 808 nm laser across a range of laser power densities, making it more appropriate for PTT in the treatment of deep‐seated tumors. To further validate the superior photothermal effect of the NIR‐II laser in deep‐seated tissues, FBDFDPA NPs were irradiated with lasers of 1060 and 808 nm at different tissue thicknesses. As illustrated in Figure 2k, at equivalent penetration depths, the temperature increases of FBDFDPA NPs subjected to 1060 nm photoirradiation were marginally greater than that observed under 808 nm photoirradiation. These results highlighted the superior tissue penetration depth and elevated maximum permissible exposure associated with the 1060 nm laser, underscoring its potential for deep‐seated tumor PTT.

### 2.3 Biocompatibility and In Vitro Photothermal Therapy

**Figure 3:**
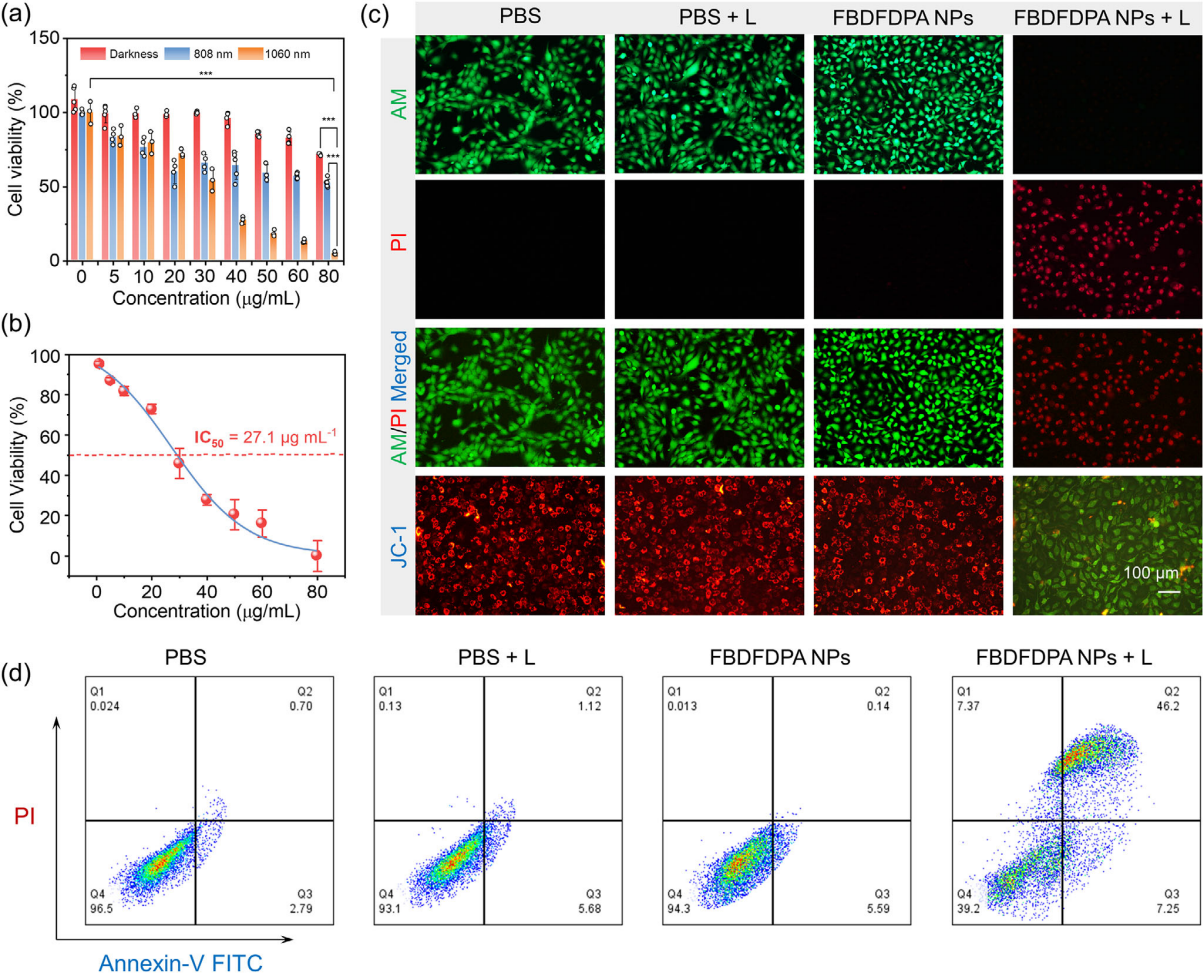
a) Viability of 4T1 cells incubated with FBDFDPA NPs under laser irradiation (1060 nm, 1.0 W cm^−2^ or 808 nm, 0.33 W cm^−2^) and dark. Error bars, mean ± s.d. (n ≥ 3). ***p < 0.001. b) IC_50_ determination of FBDFDPA NPs. c) Live/dead assay results and fluorescence microscopy images of 4T1 cancer cells with various treatments. d) Cell apoptosis of 4T1 cells examined by flow cytometry.

Motivated by the excellent photophysical properties and photothermal efficacy of FBDFDPA NPs, we continued to evaluate the biocompatibility and in vitro phototherapeutic efficacy of FBDFDPA NPs via 3‐(4,5‐dimethyl‐2‐thiazolyl) ‐2,5‐diphenyl‐2H‐tetrazolium bromide (MTT) assays. The survival rates of mouse breast cancer (4T1) cells cultured with various concentrations of FBDFDPA NPs under different laser excitations were measured. As presented in **Figure** **3**a, the viability of FBDFDPA NPs treated 4T1 cells demonstrated no obvious variation without irradiation, and the viability of FBDFDPA NPs coincubated cells was maintained at over 80% even at a concentration of 80 µg mL^−1^. In addition, there was a negligible decrease in the human normal cells (HaCat) viability after incubation with different concentrations of FBDFDPA NPs, suggesting the favorable biocompatibility of FBDFDPA NPs (Figure S13, Supporting Information). Nevertheless, following a 5‐min exposure to laser irradiation at wavelengths of 1060 nm (1.0 W cm^−2^) or 808 nm (0.33 W cm^−2^), FBDFDPA NPs showed concentration‐dependent phototoxicity (Figure 3a). Under the safety‐compliant power densities, FBDFDPA NPs exhibited stronger photothermal‐induced phototoxicity, when exposed to 1060 nm laser irradiation compared to 808 nm irradiation. The half‐maximal inhibitory concentration (IC_50_) of FBDFDPA NPs was established to be 27.1 µg mL^−1^ when subjected to 1060 nm laser photoirradiation (Figure 3b).

To validate the advantage of NIR‐II PTT, tissue penetration experiments were performed using chicken breast irradiated with 808 nm and 1060 nm lasers (Figure S14, Supporting Information). Despite the photothermal ablation efficacy of FBDFDPA NPs decreased with increasing penetration depth, cell viability under 1060 nm irradiation (1.0 W cm^−2^) remained significantly lower than that under 808 nm irradiation at the same power density. This result demonstrated the superior performance of NIR‐II PTT over NIR‐I modality for treating deep‐seated tumors.

To further assess the therapeutic potential of FBDFDPA NPs, the live/dead staining assay was conducted, wherein the green and red fluorescence signals corresponded to live and dead cells, respectively. As illustrated in Figure 3c, substantial green fluorescence was observed from the cells in the PBS group, PBS + Laser group, and FBDFDPA NPs group, suggesting that there was minimal cytotoxicity under these conditions. The cells treated with FBDFDPA NPs in conjunction with 1060 nm laser irradiation exhibited abundant red fluorescence, suggesting the remarkable phototherapeutic efficacy of FBDFDPA NPs in inducing cytotoxicity in 4T1 cells upon laser irradiation.

The decrease in mitochondrial membrane potential (MMP) serves as a critical marker for early‐stage apoptosis.^[^ ^26^ ^]^ To evaluate alterations in MMP, the JC‐1 probe was employed for analysis. Only the cells from the FBDFDPA NPs + L group showed obvious green fluorescence, while 4T1 cells in the other groups presented red fluorescence, indicating mitochondrial dysfunction and subsequent induction of apoptosis upon photoirradiation (Figure 3c). Annexin V‐FITC/PI detection assay was also conducted on tumor cells to elucidate the tumoricidal capability after different treatments. As shown in Figure 3d, in the FBDFDPA NPs + L group, ≈53.45% of 4T1 cells showed apoptosis. In contrast, minimal cell apoptosis was observed in the other three groups, reaffirming the potent photothermal therapeutic effect of FBDFDPA NPs upon NIR‐II laser activation.

### 2.4 In Vivo PAI‐Guided Antitumor Therapy

**Figure 4:**
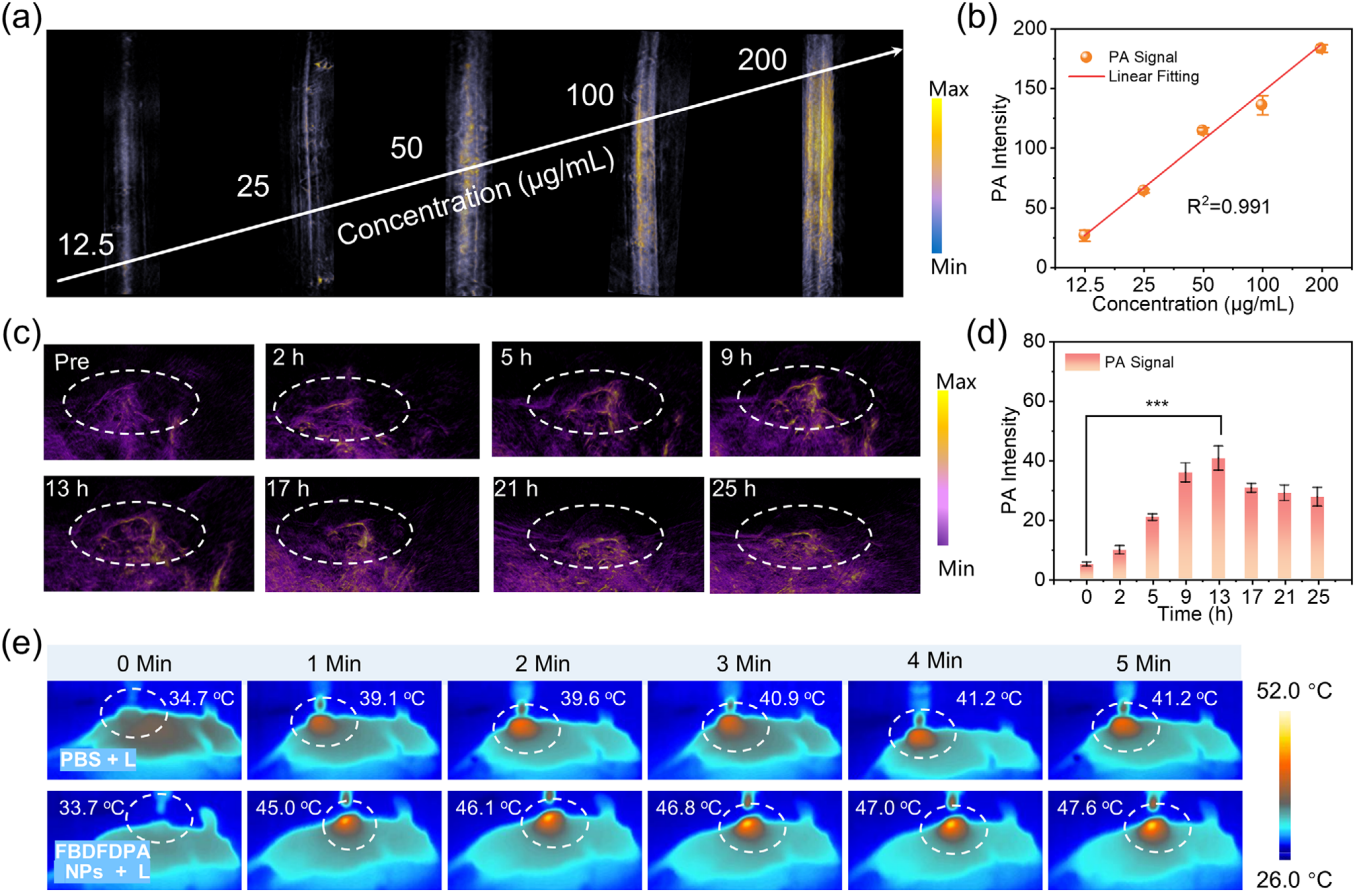
a) Photoacoustic images of FBDFDPA NPs at different concentrations. b) Linear relationship of photoacoustic intensities against the concentration of FBDFDPA NPs. Error bars, mean ± s.d. (n = 3). c) In vivo PAI of tumor sites upon 1060 nm excitation at different time spots after the injection of FBDFDPA NPs. d) In vivo photoacoustic intensity at different time spots after the injection of FBDFDPA NPs. Error bars, mean ± s.d. (n = 3). e) Thermal images of the mice under 1060 nm irradiation.

Based on the excellent photothermal performance of FBDFDPA NPs, their potential for PAI was explored. As demonstrated in **Figure** **4**a, photoacoustic images of FBDFDPA NPs were acquired at varying concentrations (0–200 µg mL^−1^) under 1060 nm laser excitation. Consistent with its excellent photothermal effect, the PA signal intensities of FBDFDPA NPs exhibited a concentration‐dependent enhancement, and a linear positive correlation between PA intensities and concentrations (0–200 µg mL^−1^) was obtained (Figure 4b). These results confirmed the promising capability of FBDFDPA NPs for in vivo NIR‐II PAI under 1060 nm laser triggering.

For NIR‐II photoacoustic imaging, the 4T1 breast tumor‐bearing mice were administered with FBDFDPA NPs (400 µg mL^−1^, 200 µL) through the tail vein. As illustrated in Figure 4c, prior to the intravenous administration of FBDFDPA NPs, the tumor region exhibited a weak PA signal due to the background signal generated by the inherent hemoglobin and melanin.^[^ ^27^ ^]^ Encouragingly, the PA signal of tumor tissues exhibited a progressive enhancement following the intravenous injection of FBDFDPA NPs, indicating selective accumulation of FBDFDPA NPs at the tumor site. 13 h following the administration of FBDFDPA NPs, the average PA signal of the tumor region reached its maximum levels, exhibiting ≈7.6‐fold increase in PA intensity compared to the pre‐injection level (Figure 4d). Then, the PA signal at the tumor site progressively decreased, indicating the elimination of FBDFDPA NPs from mice.

Due to their superior photothermal performance, FBDFDPA NPs were expected to exhibit considerable photothermal effects on tumors when excited by a 1060 nm laser. As illustrated in Figure 4e, real‐time temperature observed by an infrared camera indicated that at 13 h post‐administration of FBDFDPA NPs, the tumor site temperature exhibited a rapid increase from 33.7 to 45.0 °C within 1 min following exposure to 1060 nm laser irradiation. When the exposure duration was extended to 5 min, the tumor temperature reached 47.6 °C, which met the temperature threshold required for tumor PTT.

**Figure 5:**
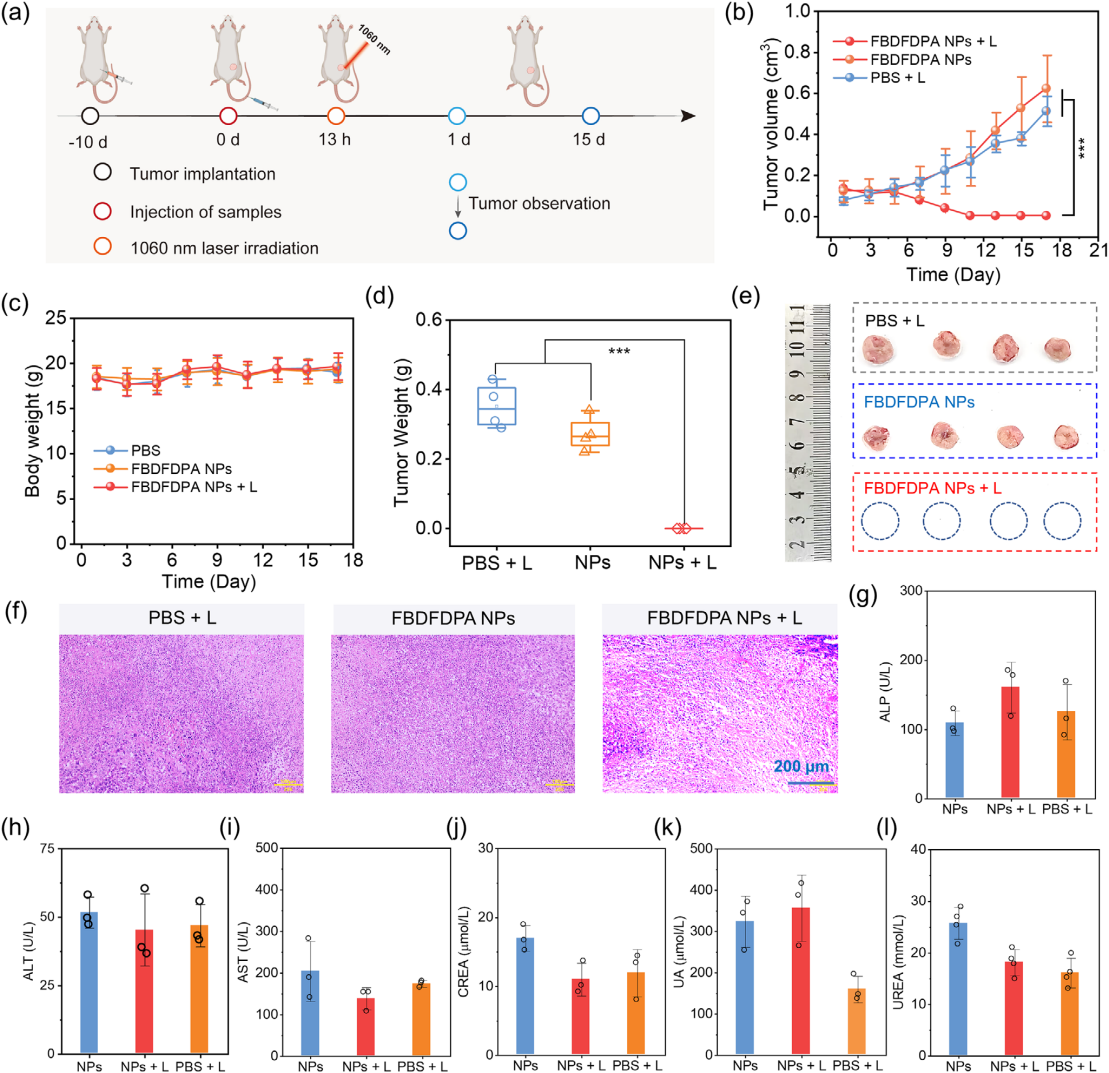
a) Schematic illustration of the schedule for tumor implantation, injection of FBDFDPA NPs, and 1060 nm laser irradiation. b) Tumor volume curves of tumor‐bearing mice during treatment. Error bars, mean ± s.d. (n = 4), ***p < 0.001. c) Body weight changes of the mice after different treatments. d) The tumor weights and e) the tumor photograph of mice after treatments. Error bars, mean ± s.d. (n = 4), ^***^ *p* < 0.001. f) H&E staining of tumor tissues after different treatments. g‐l) Serum biochemical tests. Error bars, mean ± s.d. (n = 3).

Subsequently, 4T1 tumor‐bearing mice were employed as the model to investigate the phototherapeutic effect of NIR‐II PTT (**Figure** **5**a). The mice were randomly divided into three groups for different treatments: (I) PBS with 1060 nm laser irradiation (PBS + L), (II) FBDFDPA NPs, and (III) FBDFDPA NPs with 1060 nm laser irradiation (FBDFDPA NPs + L). The absolute tumor volumes of mice were monitored during the treatment period to investigate the in vivo photothermal antitumor efficacy. The tumor volumes exhibited a rapid and continuous increase over time in both the PBS + L group and the FBDFDPA NPs group. In contrast, the tumor growth in the FBDFDPA NPs + L group was significantly inhibited, and significant tumor elimination was achieved (Figure 5b,d,e). It was noteworthy to mention that no instances of tumor recurrence were observed during the treatment period. Furthermore, only minor scarring was observed at the treatment site at 15 days post‐therapy (Figure S15, Supporting Information). This strongly supported the superior anti‐tumor efficacy of FBDFDPA NPs under the 1060 nm laser irradiation. Furthermore, the body weight of the mice was monitored at two‐day intervals to assess the safety profile of the treatment. All groups of mice exhibited similar body weight patterns during the treatment period, indicating the good biosafety profile of FBDFDPA NPs (Figure 5c). Additionally, histological analysis of the resected tumor tissues was carried out using hematoxylin and eosin (H&E) staining. Tumor H&E images of the FBDFDPA NPs + L group showed significant tissue damage, further demonstrating the excellent therapeutic efficacy of FBDFDPA NPs when subjected to the NIR‐II laser irradiation (Figure 5f).

To further evaluate the biosafety of FBDFDPA NPs, we carried out H&E staining on the key organs (heart, liver, spleen, lungs, and kidneys), in addition to performing a routine blood examination. H&E staining revealed that the mice did not display any notable histological damage or pathological abnormalities in their vital organs throughout the treatment course (Figure 5g–l; Figure S16, Supporting Information). The comprehensive biochemical analysis of blood revealed that all relevant biochemical parameters fall within the normal range, indicating the in vivo biosafety of FBDFDPA NPs. The results indicated that FBDFDPA NPs represented a promising and efficient option for NIR‐II PTT.

## 3 Conclusion

In summary, we have successfully synthesized a D‐A‐D structured NIR‐II absorbing boron difluoride formazanate dye FBDFDPA for NIR‐II PAI‐guided PTT through a rational donor engineering strategy. By replacing the alkoxy‐substituted TPA donor unit with the alkoxy‐substituted DPA unit, FBDFDPA presented enhanced π‐skeleton planarity and minimized steric hindrance. This structural modification boosted ICT efficiency, promoted a delocalized charge transfer state, and ultimately induced a 138 nm redshift in the absorption spectrum, extending its band into the NIR‐II region. Encapsulated with Pluronic F‐127, FBDFDPA NPs demonstrated excellent NIR‐II absorption (ε = 7.66 × 10^3^ M^−1^ cm^−1^), exceptional photostability, and high photothermal conversion efficiency (*η* = 54.4%). In 4T1 tumor‐bearing mice, FBDFDPA NPs enabled 1060 nm laser‐triggered NIR‐II PAI‐guided PTT, achieving complete tumor ablation and demonstrating negligible systemic toxicity. This work highlights the importance of the planarization of the conjugated skeleton for the design of NIR‐II dyes.

## 4 Experimental Section

### Instruments and Characterization

The size and scale were characterized with transmission electron microscopy (TEM, JEM‐2010FEF) and dynamic light scattering (DLS, Malvern Zeta Sizer). The temperature of these solution samples was recorded using an infrared thermal imaging camera (E50, Arlington) for photothermal performance analysis. The cells' fluorescence imaging was detected by an inverted fluorescence microscope (Nikon ECLIPSE Ts2R). The PA signal of the tumor was observed under a multispectral PA tomography instrument (TomoWave LOIS‐3D, TomoWave Laboratories, Inc.). The density functional theory (DFT) calculations were performed by employing the DFT‐B3LYP function and the B3LYP/def2‐SVP basis set, as implemented in the Gaussian 09 program package. Electrostatic potential (ESP) distributions of FBDFTPA and FBDFDPA were generated with structures optimized by Gaussian 09, and ESP was calculated using Multiwfn 3.8 and VMD. All experimental animal procedures were performed with approval from the Institutional Animal Care and Use Committee of Zhejiang Provincial People's Hospital (approval number: 20 231 201 154 816 132 791).

### Preparation of the Nanoparticles

The FBDFDPA NPs and FBDFTPA NPs were prepared via the nanoprecipitation method. To prepare the NPs, FBDFDPA/FBDFTPA (1 mg) and F‐127 (10 mg) were dissolved in tetrahydrofuran (THF, 1.0 mL) and mixed uniformly. The mixed solution was rapidly added to 10 mL of deionized water and subjected to sonication for 30 min. Following the complete evaporation of the organic solvent THF, nanoparticles were successfully obtained.

### In Vivo NIR‐II PAI and Photothermal Imaging

For NIR‐II photoacoustic imaging, the 4T1 breast tumor‐bearing mice were administered with FBDFDPA NPs (400 µg mL^−1^, 200 µL) through the tail vein. Then, in vivo PAI was carried out on the commercial optoacoustic imaging system at designated time intervals after intravenous injection of FBDFDPA NPs. For in vivo photothermal imaging, the infrared thermal images of mice were acquired using an IR camera during the irradiation of 1060 nm laser (1.0 W cm^−2^) at 13 h post‐administration with FBDFDPA NPs (200 µL). Mice injected with PBS under the same irradiation conditions were used as the control.

### In Vivo Antitumor Efficacy

When the tumor volume reached ∼100 mm^3^, 4T1 tumor‐bearing mice were randomly assigned to different treatment groups (I: PBS + L, II: FBDFDPA NPs, III: FBDFDPA NPs + L, n = 4). The administration dose of FBDFDPA NPs was 4 mg kg^−1^. Laser irradiation (1.0 W cm^−2^, 5 min) was applied. Tumor volumes and mouse weights were assessed every other day during the following 14 days. At the end of the 14‐day treatment, the mice were euthanized, and pathological analysis of the tumors and major organs was performed. Tumor tissue sections were observed under an optical microscope.

### Statistical Analysis

Statistical analysis was performed using Origin 2024b. Data are presented as mean ± standard deviation (mean ± s.d.). Statistical differences were analyzed using t‐tests. Statistical significance was defined as follows: ^*^p < 0.05, ^**^p < 0.01, ^***^p < 0.001, and ^****^p < 0.0001.

## Conflict of Interest

The authors declare no conflict of interest.

## Supporting information



Supporting Information

## Data Availability

The data that support the findings of this study are available from the corresponding author upon reasonable request.
