# Supplementary material for: Boron Difluoride Formazanate Dye With Donor Planarization Engineering for 1060 nm Laser Activated Photothermal Theranostics
Source: Adv Sci (Weinh). 2025 Jul 6;12(37):e06226. doi: 10.1002/advs.202506226 (PMC12499441; doi:10.1002/advs.202506226)
Supplement: Supplementary file 1 — Supporting Information [file ADVS-12-e06226-s001.docx]

*Supplementary Information*

**Boron Difluoride Formazanate Dye with Donor Planarization Engineering for 1060 nm Laser Activated Photothermal Theranostics**

*Kang Xu, Mengchen Luo, Weili Wang, Tian Zhang, Yang Chen, Jinjun Shao,* Peng Chen, Xiaochen Dong,* and Yu Cai**

K. Xu, M. Luo, W. Wang, T. Zhang, J. Shao, X. Dong

State Key Laboratory of Flexible Electronics (LoFE) & Institute of Advanced Materials (IAM), School of Flexible Electronics (Future Technologies), Nanjing Tech University, Nanjing 211816, China.

E-mail: iamxcdong@njupt.edu.cn; [iamjjshao@njtech.edu.cn](mailto:iamjjshao@njtech.edu.cn)

X. Dong

School of Chemistry & Materials Science, Jiangsu Normal University, Xuzhou 221116, China.

E-mail: iamxcdong@njupt.edu.cn

P. Chen

School of Chemistry, Chemical Engineering and Biotechnology, Institute for Digital Molecular Analytics and Science, Lee Kong Chian School of Medicine, Nanyang Technological University, 62 Nanyang Drive, Singapore 637459, Singapore.

Y. Cai

Center for Rehabilitation Medicine, Rehabilitation & Sports Medicine Research Institute of Zhejiang Province, Department of Rehabilitation Medicine, Cancer Center, Zhejiang Provincial People's Hospital (Affiliated People's Hospital), Hangzhou Medical College, Hangzhou 310014, China

E-mail: [iamycai@163.com](mailto:iamycai@163.com)

**1. Synthesis procedures**

**Figure S1.** Synthesis routes of FBDFDPA and FBDFTPA.

*1.1 Synthesis of Compound* ***2***

2-Bromofluorene (13 g, 53 mmol) was dissolved in 90 mL of 1,2-dichloroethane, and then 25 ml of nitric acid was added dropwise. After stirring at 0 °C for 30 minutes, the light green solid was precipitated, filtered, and dried to obtain compound **2** (14 g, 91% yield).

^1^H NMR (400 MHz, CDCl_3_): δ ppm 8.40 (s, 1H, Ar-H), 8.31 (dd, *J* = 8.6, 1.8 Hz, 1H, Ar-H), 7.85 (d, *J* = 8.6 Hz, 1H, Ar-H), 7.77 (d, *J* = 1.2 Hz, 1H, Ar-H), 7.73 (d, *J* = 7.9 Hz, 1H, Ar-H), 7.60-7.57 (m, 1H, Ar-H), 4.01 (s, 2H, CH_2_).

*1.2 Synthesis of Compound* ***3***

KOH (10.000 g, 178 mmol) was added to 70 mL DMSO and stirred for 10 min. Then, compound **2** (10.000 g, 34 mmol) was added and reacted overnight at room temperature. After the reaction was complete, the solution was extracted with ethyl acetate and concentrated in a vacuo to give the crude product. Purification by silica gel column chromatography (eluent: petroleum ether) gave the purified light-yellow product (15.200 g, 80% yield).

^1^H NMR (400 MHz, CDCl_3_): δ ppm 8.27 (dd, *J* = 8.6, 1.8 Hz, 1H, Ar-H), 8.19 (d, *J* = 2.4 Hz, 1H, Ar-H), 7.78 (d, *J* = 8.6 Hz, 1H, Ar-H), 7.65 (d, *J* = 7.3 Hz, 1H, Ar-H), 7.55-7.53 (m, 2H, Ar-H), 2.16-2.01 (m, 4H, CH_2_), 0.32 (t, J = 7.3 Hz, 6H, CH_3_).

*1.3 Synthesis of Compound* ***4***

Compound **3** (7.00 g, 20 mmol), iron powder (3.38 g, 61 mmol), and NH_4_Cl (2.16 g, 40 mmol) were added to a mixed solvent (110 mL of ethanol and 30 mL of water) and refluxed at 85 ℃ for 3 h under nitrogen atmosphere. The mixture was filtered and extracted with dichloromethane. The solvent was removed and the crude product was purified by silica gel chromatography (petroleum ether: CH_2_Cl_2_ = 1:1 (*v*:*v*)) to afford a white solid (5.80 g, 92% yield).

^1^H NMR (400 MHz, CDCl_3_): δ ppm 7.45 (d, *J* = 7.9 Hz, 1H, Ar-H), 7.40-7.37 (m, 3H, Ar-H), 6.68 (dd, *J* = 7.9, 1.8 Hz, 1H, Ar-H), 6.65 (d, *J* = 2.4 Hz, 1H, Ar-H), 4.05 (s, 2H, NH_2_), 1.99-1.89 (m, 4H, CH_2_), 0.33 (t, *J* = 7.3 Hz, 6H, CH_3_).

*1.4 Synthesis of Compound* ***5***

*Step 1*:

Cyanoacetic acid (134 mg, 1.6 mmol) and sodium hydroxide (0.800 g, 20 mmol) were dissolved in deionized water (50 mL), and cooled to 0 °C. Compound 3 (1.000 g, 3 mmol) was added to deionized water (5 mL) and cooled to 0 °C in the air. Then, concentrated hydrochloric acid (1.2 mL, 14 mmol) was added dropwise. The solution was cooled to 0 °C and stirred for 30 min. Afterward, a cooled solution of sodium nitrite (NaNO_2_, 250 mg, 3.6 mmol) in deionized water (5 mL) was added to the initial solution and stirred at 0 °C for 5 min. The diazonium salt solution was added dropwise to the alkaline solution and stirred at 0 °C for 2 h. The mixture was filtered to give a red solid. Purified through silica gel chromatography (petroleum ether: ethyl acetate = 30:1 (*v*:*v*)) to give a purple solid.

*Step 2*:

Under a nitrogen atmosphere, the purple solid in *step 1* and triethylamine (1 mL) were added to anhydrous toluene (20 mL). Then, Boron trifluoride diethyl etherate (1 mL) was slowly added to the above solution, and the mixture was heated to 85 °C and stirred overnight. Subsequently, the mixture was cooled to room temperature, and deionized water was added to the solution to quench the reaction. The mixture was extracted with dichloromethane and concentrated to obtain the crude product. The crude product was purified by silica gel column chromatography (petroleum ether: ethyl acetate = 1:20 (*v*:*v*)) to afford a blue solid (100 mg, 5% yield)

^1^H NMR (400 MHz, CDCl_3_): δ ppm 7.98 (d, *J* = 7.9 Hz, 2H, Ar-H), 7.85 (s, 2H, Ar-H), 7.77 (d, *J* = 8.6 Hz, 2H, Ar-H), 7.62 (d, *J* = 8.6 Hz, 2H, Ar-H), 7.54-7.50 (m, 4H, Ar-H), 2.17-2.01 (m, 8H, CH_2_), 0.34 (t, *J* = 7.3 Hz, 12H, CH_3_).

*1.5 Synthesis of FBDFTPA*

Compound **5** (100 mg, 0.13 mmol), 4-Methoxy-N-(4-MethoxyPhenyl)-N-(4-(4,4,5,5- Tetramethyl-1,3,2-Dioxaborolan-2-yl) Phenyl) Aniline (150 mg, 0.52 mmol), K_2_CO_3_ (15 mg, 0.104 mmol), Pd(PPh_3_)_4_ (15 mg, 0.013 mmol) were added to 40 ml mixed solvent of water and toluene (v/v = 1/3). Under a nitrogen atmosphere, the mixture was heated to 85 °C and stirred overnight. Subsequently, the mixture was cooled to room temperature and the solvent was removed. The crude product was purified by silica gel column chromatography in 10:1 petroleum ether/ethyl acetate to afford a blue solid.

^1^H NMR (400 MHz, CDCl_3_): δ ppm 7.99 (d, *J* = 8.6 Hz, 2H, Ar-H), 7.88 (s, 2H, Ar-H), 7.78 (d, *J* = 8.6 Hz, 2H, Ar-H), 7.59 (d, *J* = 7.9 Hz, 2H, Ar-H), 7.54-7.49 (m, 4H, Ar-H), 7.11-7.01 (m, 14, Ar-H), 6.87-6.82 (m, 10H, Ar-H), 3.81 (s, 12H,O-CH_3_), 2.18-2.08 (m, 8H, CH_2_), 0.38 (t, *J* = 7.3 Hz, 12H, CH_3_).

^13^C NMR (100 MHz, CDCl_3_): δ ppm 155.9, 151.9, 151.8, 148.4, 145.0, 142.4, 141.5, 140.7, 138.3, 135.7, 132.7, 127.6 , 127.1, 126.6, 123.3, 121.1, 120.9, 120.6, 120.1, 118.6, 117.3, 114.7, 56.7, 55.5, 32.7, 24.8, 8.6.

MALDI-TOF Mass (m/z): Calcd for C_76_H_68_BF_2_N_7_O_4_ [M]^+^: 1192.5472, found 1192.2534.

*1.6 Synthesis of FBDFDPA*

Under a nitrogen atmosphere, compound **4** (100 mg, 0.13 mmol), Bis-(4-methoxyphenyl)-amine (119 mg, 0.52 mmol), Pd(OAc)_2_ (23 mg, 0.1 mmol), P(*t*-Bu)_3_·HBF_4_ (8 mg, 10 mmol), *t*-BuONa (25 mg, 0.26 mmol) were added to 20 mL toluene. The mixture was heated to 85 °C and stirred overnight. Then, the reaction mixture was cooled to room temperature, quenched with saturated aq NH_4_Cl, and extracted with ethyl acetate. The solvent was removed and the crude product was purified by silica gel chromatography.

^1^H NMR (400 MHz, CDCl_3_) δ ppm 7.92 (d, *J* = 8.6 Hz, 2H, Ar-H), 7.79 (s, 2H, Ar-H), 7.61 (d, *J* = 8.6 Hz, 2H, Ar-H), 7.51 (d, *J* = 7.9 Hz, 2H, Ar-H), 7.09 (d, *J* = 8.6 Hz, 8H, Ar-H), 6.92-6.84 (m, 12H, Ar-H), 3.82 (s, 12H, O-CH_3_), 2.05-1.83 (m, 8H, CH_2_), 0.37 (t, *J* = 7.3 Hz, 12H, CH_3_).

^13^C NMR (100 MHz, CDCl_3_): δ ppm 156.1, 152.9, 151.1, 149.9, 145.2, 141.6, 140.7, 132.5, 126.7, 123.2, 121.4, 119.8, 119.0, 116.9, 114.7, 56.3, 55.5, 32.5, 29.7, 8.5.

MALDI-TOF Mass (m/z): Calcd for C_64_H_61_BF_2_N_7_O_4_ [M]^+^:1040.4846, found 1039.6385.

**2. Experimental Section**

*Instruments and Characterization:* ^1^H and ^13^C NMR spectra were measured on a JEOL spectrometer (JNM-ECZ400S, 400 MHz Japan). Matrix-assisted laser desorption ionization time-of-flight (MALDI-TOF) mass spectroscopy was performed on a Bruker MALDI-TOF. UV-vis absorbance spectrum was recorded with a UV-3600 Shimadzu UV-vis-NIR spectrometer.

*Photostability evaluation:* FBDFDPA NPs and ICG were continuously irradiated with lasers (1060 nm at 1.0 W cm^-2^ and 808 nm at 1.0 W cm^-2^, respectively) for 10 min, and the absorption spectra were recorded regularly to evaluate their photostability.

*Photothermal performance measurement:* FBDFDPA NPs and FBDFTPA NPs with concentrations of 100, 50, 25, and 12.5 μg mL^-1^, were continuously irradiated for 10 min under 1060 nm and 808 nm laser with the power density of 1.0 W cm^-2^, respectively, and deionized water was used as a reference under the same conditions. The photothermal properties of FBDFDPA NPs solution with a concentration of 50 μg mL^-1^ at power densities of 1.0, 0.8, 0.6, 0.4, and 0.2 W cm^-2^ were investigated using deionized water as a reference. The infrared thermal camera records the temperature every 30 seconds.

*Calculation of photothermal conversion efficiency:* In addition, the photothermal conversion efficiency (*η*) of FBDFDPA NPs and FBDFTPA NPs in the aqueous solution was calculated according to the reported literature. Briefly, FBDFDPA NPs solution (50 μg mL^-1^) was exposed to a 1060 nm laser at the power density of 1.0 W cm^-2^ for 10 minutes and cooled down for 10 minutes. FBDFTPA NPs were exposed to an 808 nm laser at the power density of 1.0 W cm^-2^. The temperature was recorded during the whole heating-cooling process.

*ƞ* = (*hs*∆*T*_max_ − *Q*_s_)/*I*(1 − 10^-A^)

*τ*_s_ = m_D_C_D_/*hs*

*Q*_s_ = C_D_*∆T*_max_/*t*

in which, *∆T*_max_ represents the highest temperature variation observed after irradiating the aqueous solution of FBDFDPA NPs for 10 minutes, and *τ*_s_ refers to the time constant of the sample system. The *m_D_* represents the mass of deionized water, which in this case is 1.0 g. The *C_D_* represents the heat capacity of deionized water, which is 4.2 J g^-1^. *Q*_s_ represents heat dissipated from the laser mediated by the solvent and container. The *I* is the laser power density. The A is the absorbance of FBDFDPA NPs aqueous solution at 1060 nm.

FBDFTPA NPs were exposed to 808 nm laser at the power density of 1.0 W cm^-2^, and The A is the absorbance of FBDFTPA NPs aqueous solution at 808 nm, Other experimental parameters remain unchanged.

*Cell Culture:* 4T1 cells were obtained from the School of Pharmaceutical Sciences, Nanjing Tech University. 4T1 cells were cultured in 1640 medium, which contained 1% (v/v) antibiotics and 10% (v/v) fetal bovine serum (FBS) in a humidified 37 °C atmosphere containing 5% CO_2_.

*MTT Assay:* The 4T1 cells were seeded at a density of 5 × 10^3^ cells/well in a 96-well plate and incubated for 24 h. Subsequently, the cells were treated with various concentrations of FBDFDPA NPs in a fresh culture medium. After 12 h co-incubation, the cells are exposed to 808 nm or 1060 nm laser irradiation (1.0 W cm^-2^) for 5 min. Meanwhile, cells coincubated with FBDFDPA NPs without laser irradiation were also conducted for the dark cytotoxicity study. Then, MTT (5 mg mL^-1^, 20 μL) dissolved in PBS was added into each well, followed by an additional 4 hours of cultivation. Finally, the medium was removed, DMSO (200 µL) was added, and measurements of absorbance were subsequently recorded by a microplate reader.

*Live-dead cell staining:* 4T1 cells were seeded onto 6-well plates at a density of 1 × 10^5^ cells per well and cultured in 1.0 mL of 1640 media at 37 °C for 24 h. Then, fresh 1640 media containing FBDFDPA NPs (100 μg mL^-1^) were added and incubated for 4 h at 37 °C. Next, the Laser groups were exposed to a 1060 nm laser (1.0 W cm^-2^) for 5 minutes per well. Next, cells were washed with PBS three times and subsequently co-stained with Calcein AM (0.5 μM) and PI (2 μM) for 15 min at 37 °C. Finally, cells were washed with PBS twice and imaged by an FL microscope. (Calcein-AM, E_x_/E_m_: 488/510 nm; PI, E_x_/E_m_: 530/615 nm).

*Mitochondrial Integrity Assay:* 4T1 cells with a density of 1×10^5^ containing complete 1640 (with FBS) media were incubated in a 6-well plate for 24 h. The 4T1 cells were treated with fresh medium containing FBDFDPA NPs (100 μg mL^-1^) or PBS and divided into four groups: (I) FBDFDPA NPs without Laser, (II) FBDFDPA NPs + Laser, (III) PBS without Laser, (IV) PBS + Laser. The Laser group was irradiated with a 1060 nm laser (0.8 W cm^-2^) for 5 minutes per well. After incubating for 2 hours, the culture medium was replaced with a JC-1 staining solution according to the manufacturer’s protocol. Finally, the cells were imaged using a fluorescence microscope.

**3. Supplementary Figures**

**
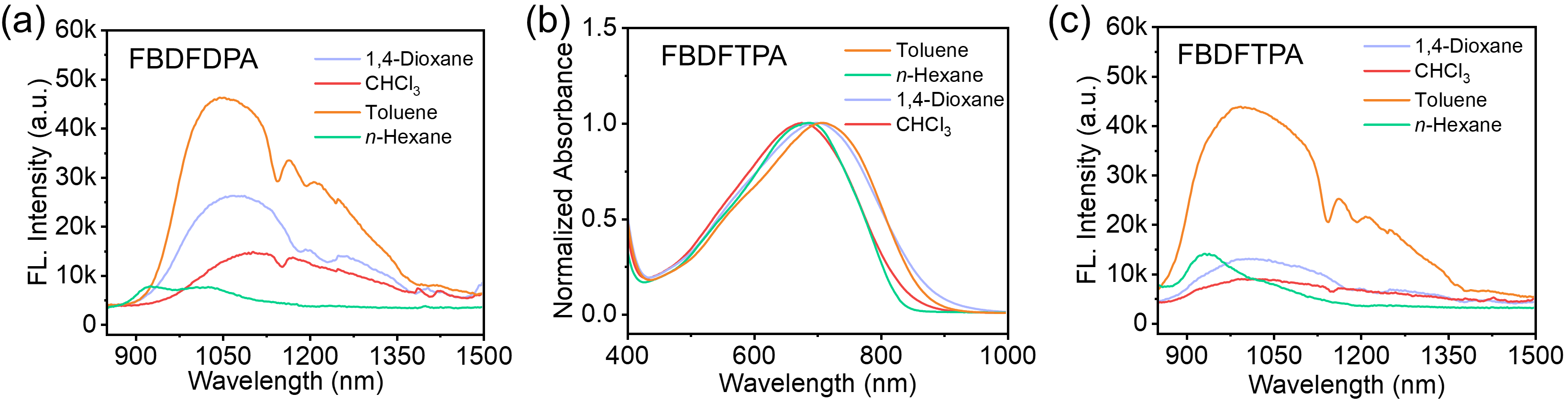
**

**Figure S2.** (a) Fluorescence spectra of FBDFDPA in 1,4-Dioxane, CHCl_3_, Toluene, and *n*-Hexane. (b) Normalized absorption, and (c) fluorescence spectra of FBDFTPA in 1,4-Dioxane, CHCl_3_, Toluene, and *n*-Hexane.

**Table S1.** Detailed absorption and emission peaks of FBDFDPA and FBDFTPA in different solvents.

| **Solvent** | **Δ*f*** | **FBDFDPA** | | |  | **FBDFTPA** | | |
| --- | --- | --- | --- | --- | --- | --- | --- | --- |
|  |  | **λ_abs_ (nm)** | **λ_em_ (nm)** | **Δ*ν* = *ν*_abs_ − *ν*_em_** |  | **λ_abs_ (nm)** | **λ_em_ (nm)** | **Δ*ν* = *ν*_abs_ − *ν*_em_** |
| *n*-Hexane | -0.048 | 778 | 924 | 2030 |  | 686 | 932 | 3848 |
| Toluene | 0.014 | 817 | 1054 | 2752 |  | 712 | 994 | 3985 |
| Dioxane | 0.025 | 792 | 1064 | 3228 |  | 698 | 1020 | 4522 |
| CHCl_3_ | 0.148 | 831 | 1172 | 3501 |  | 676 | 1032 | 5103 |

**
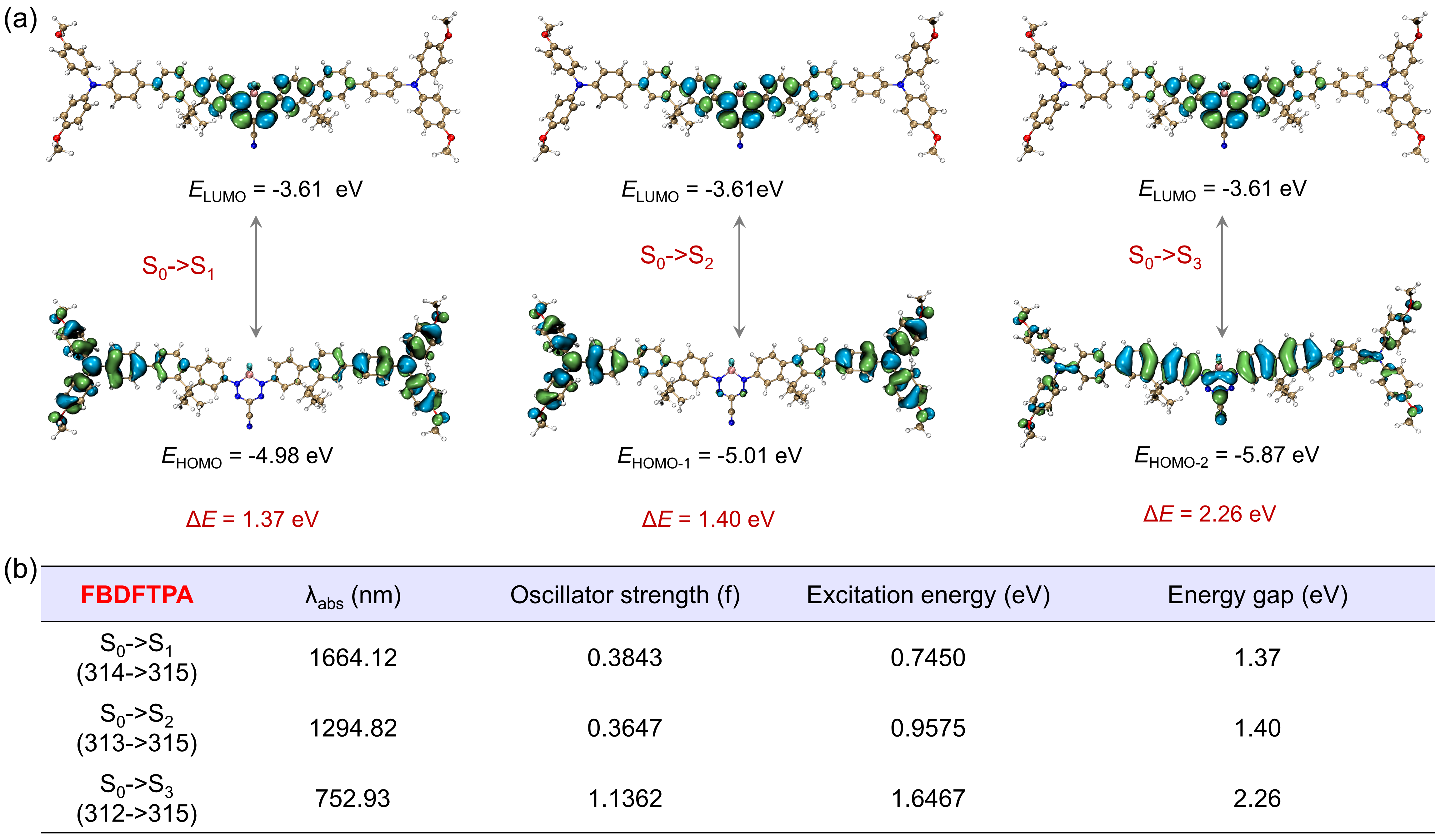
**

**Figure S3.** Calculated frontier molecular orbitals and energy levels of FBDFTPA.

**
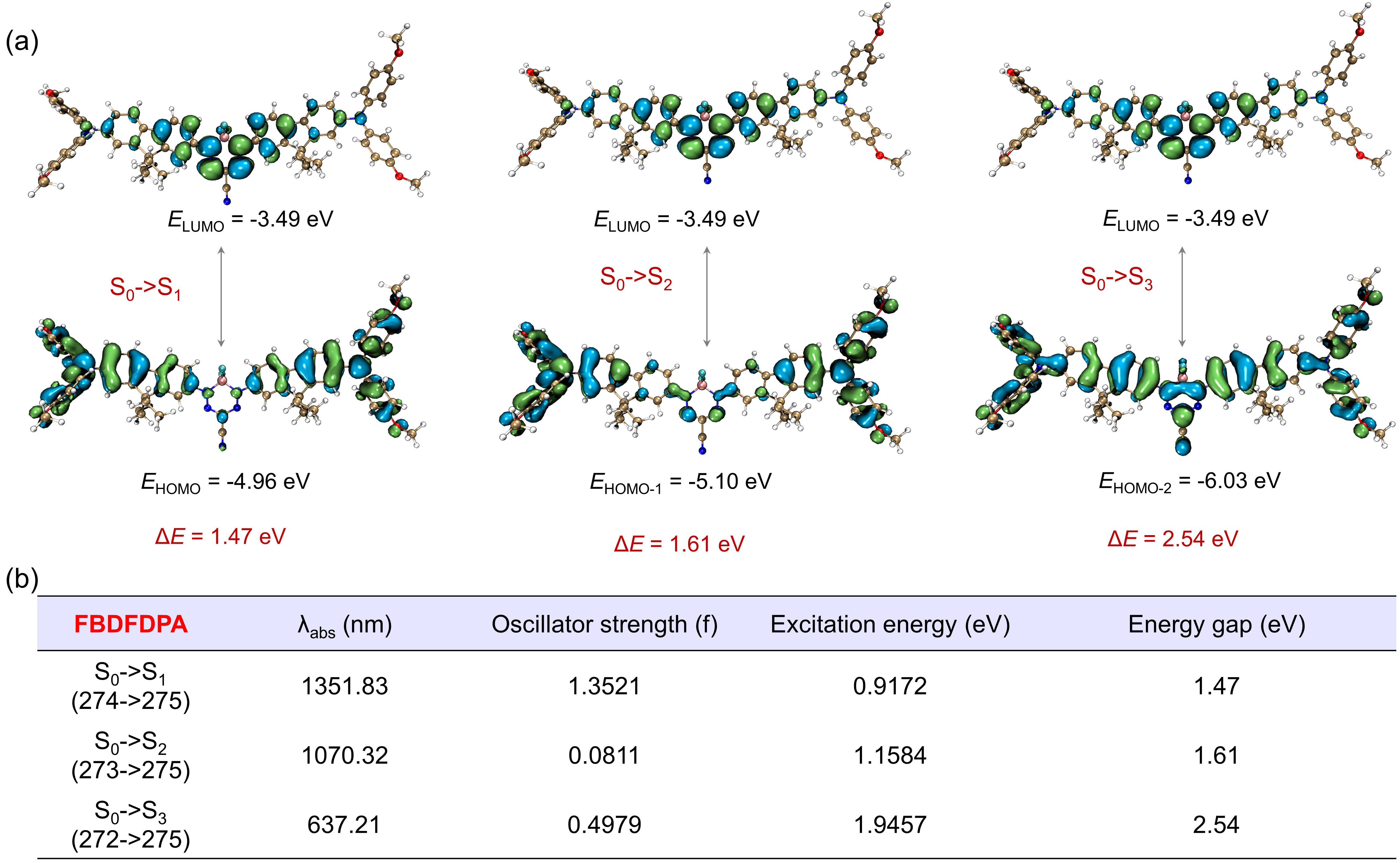
**

**Figure S4.** Calculated frontier molecular orbitals and energy levels of FBDFDPA.

**
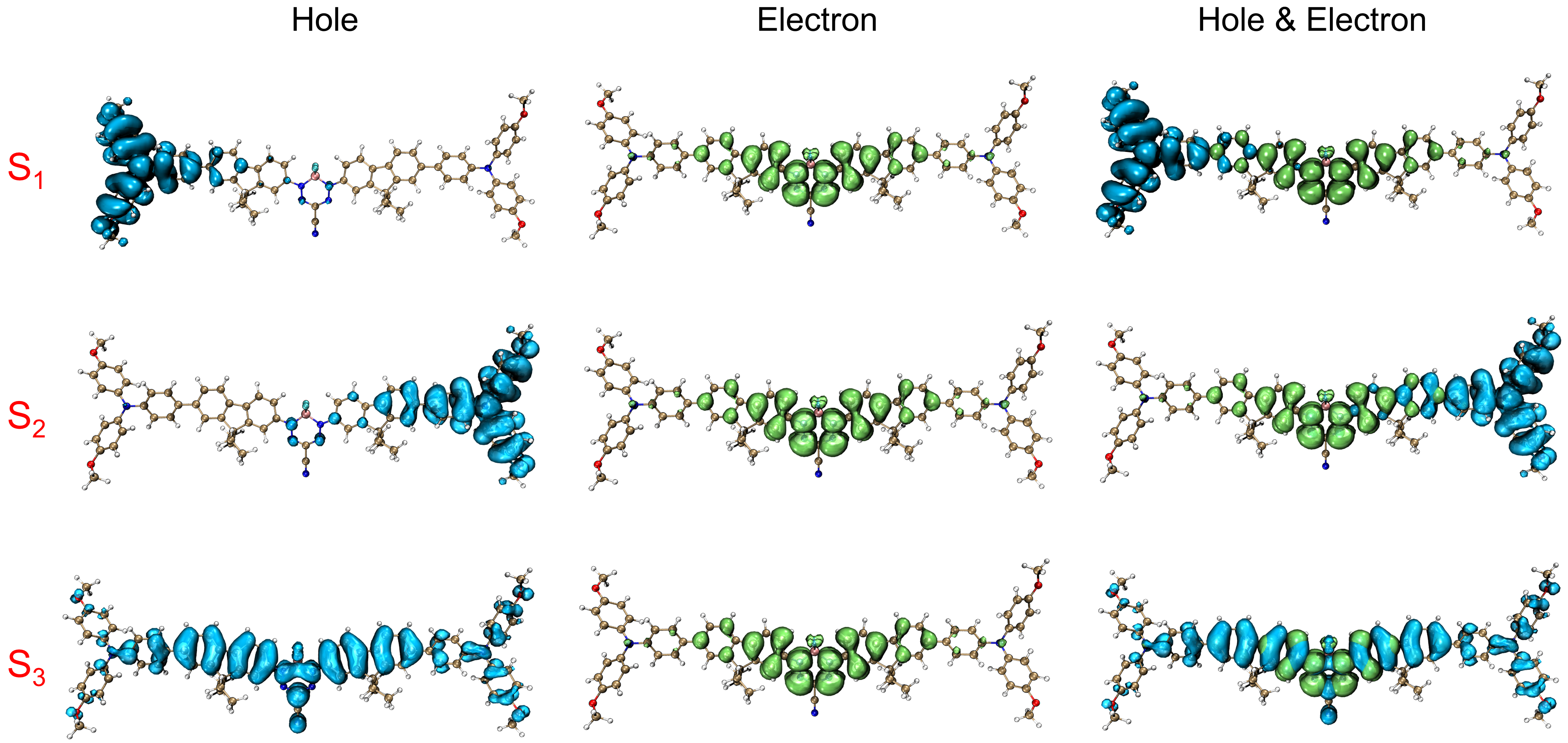
**

**Figure S5.** The Hole&Electron Graphs of FBDFTPA.

**
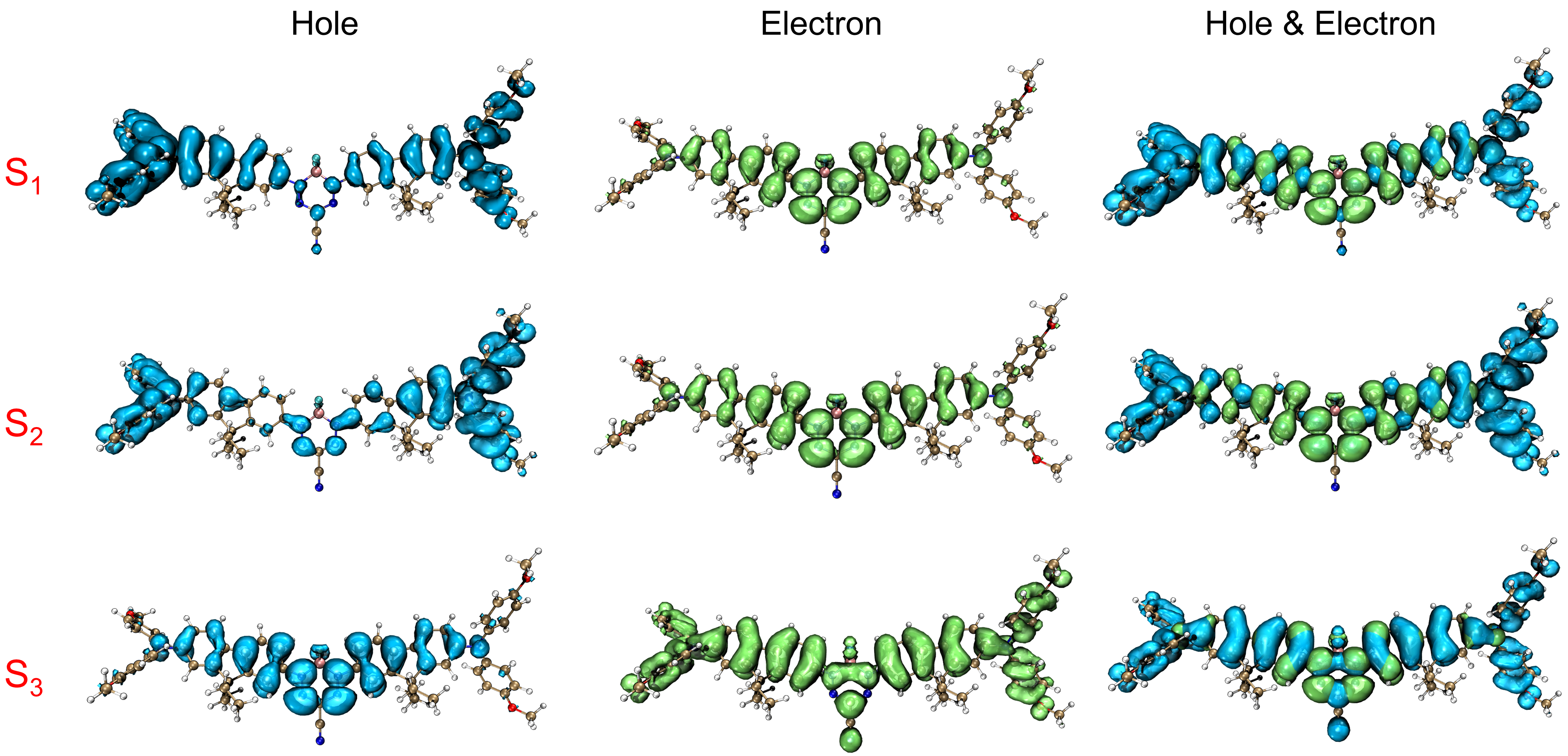
**

**Figure S6.** The Hole&Electron Graphs of FBDFDPA.

**
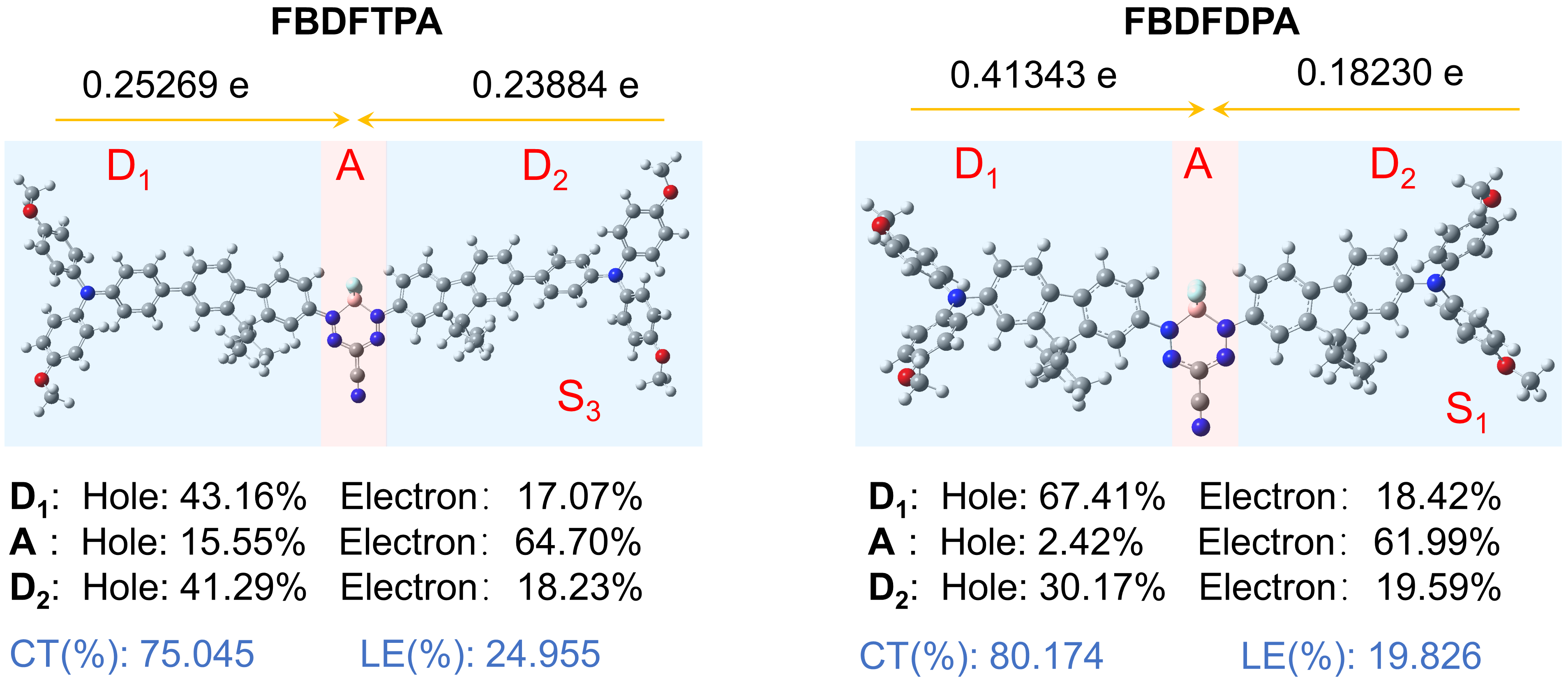
**

**Figure S7.** Diagram of the amounts of charge transfer between fragments (acceptor core and donor parts) of FBDFTPA and FBDFDPA upon vertical excitation.

**Table S2.** The summary of the computational results for FBDFTPA and FBDFDPA.

|  | **D^[a]^ (Å)** | **S_HE_^[b]^** | **H^[c]^ (Å)** | **t^[d]^ (Å)** | ***E*_coul_^[e]^ (eV)** | **HDI^[f]^** | **EDI^[g]^** | **Excitation**  **energy (eV)** | **Dipole moment**  **(a.u.)** |
| --- | --- | --- | --- | --- | --- | --- | --- | --- | --- |
| FBDFTPA  (S_0_->S_3_) | 1.318 | 0.624 | 6.207 | 0.149 | 2.877 | 4.06 | 9.74 | 1.6467 | 2.481 |
| FBDFDPA  (S_0_->S_1_) | 4.593 | 0.429 | 7.256 | 1.716 | 2.364 | 5.66 | 9.32 | 0.9172 | 8.635 |

^[a]^ The distance between centroid of hole and electron.

^[b]^ The overlap of holes and electrons.

^[c]^ The overall average distribution extent of electrons and holes.

^[d]^ The separation degree of holes and electrons.

^[e]^ Exciton binding energy.

^[f]^ Hole delocalization index.

^[g]^ Electron delocalization index.

**
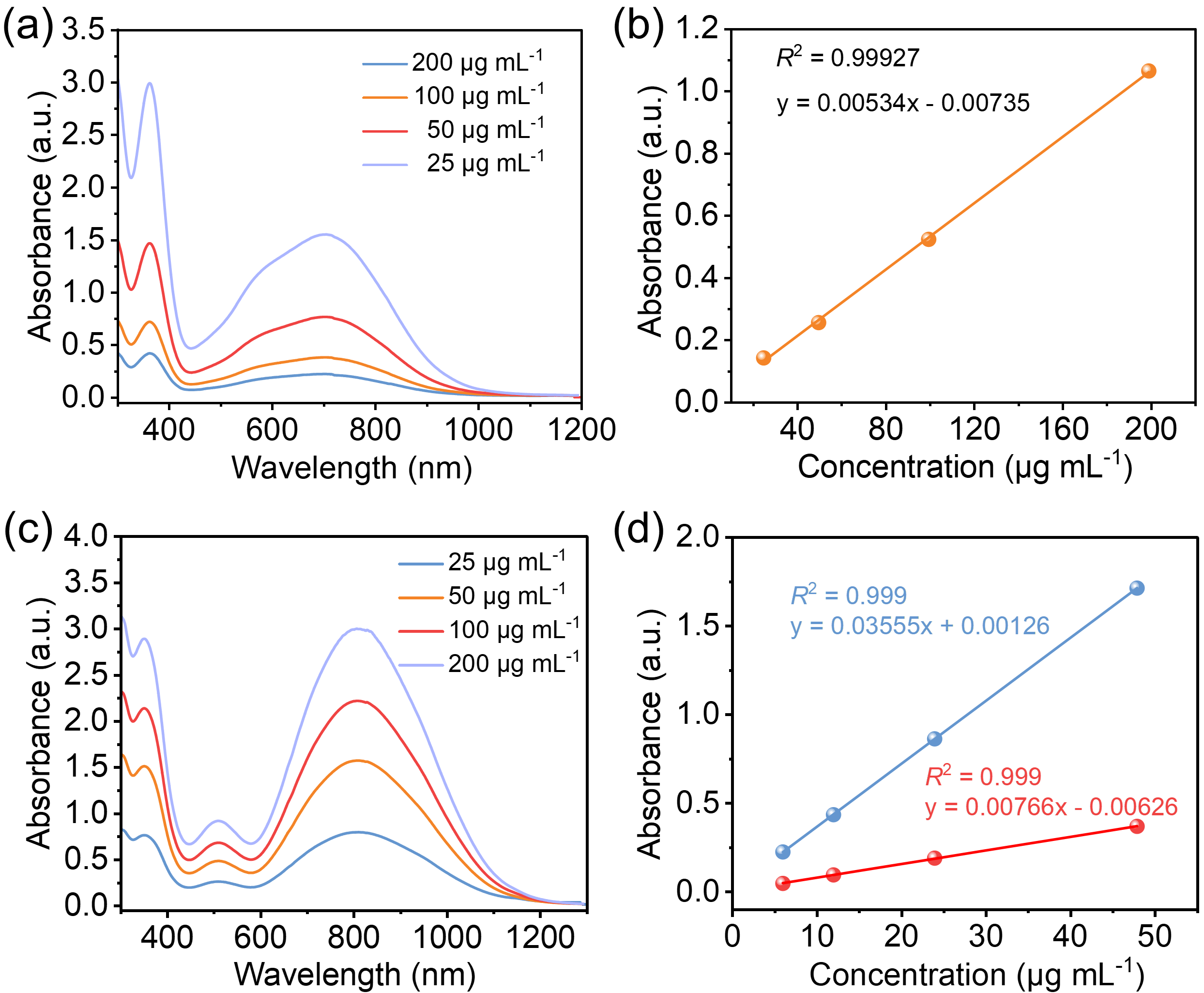
**

**Figure S8.** (a) UV-vis absorption spectra of FBDFTPA NPs at different concentrations. (b) Mass extinction coefficient of FBDFTPA NPs at 808 nm. (c) UV–vis absorption spectra of FBDFDPA NPs at different concentrations. (d) Mass extinction coefficient of FBDFDPA NPs at 808 nm and 1060 nm.

**
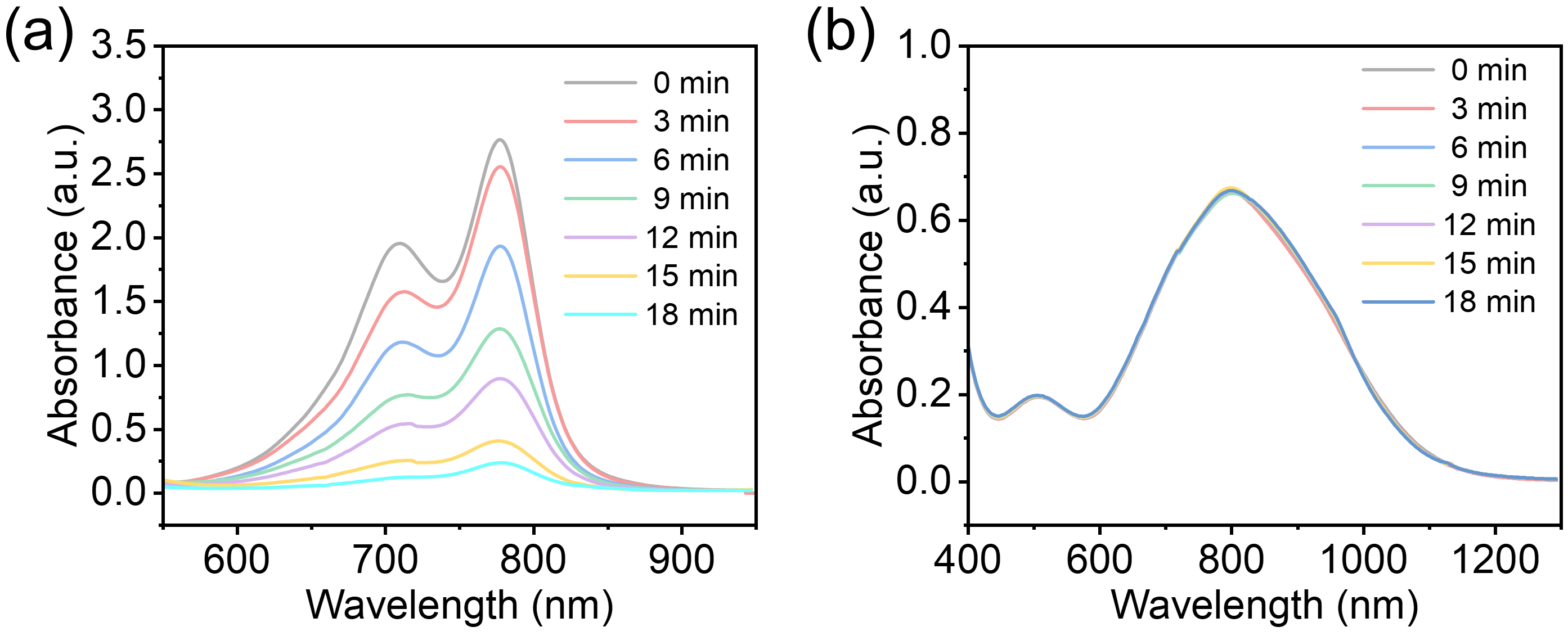
**

**Figure S9.** UV-vis absorption spectra of (a) ICG under 808 nm laser irradiation (1.0 W cm^-2^), and (d) FBDFDPA NPs under 1060 nm laser irradiation (1.0 W cm^-2^).

**
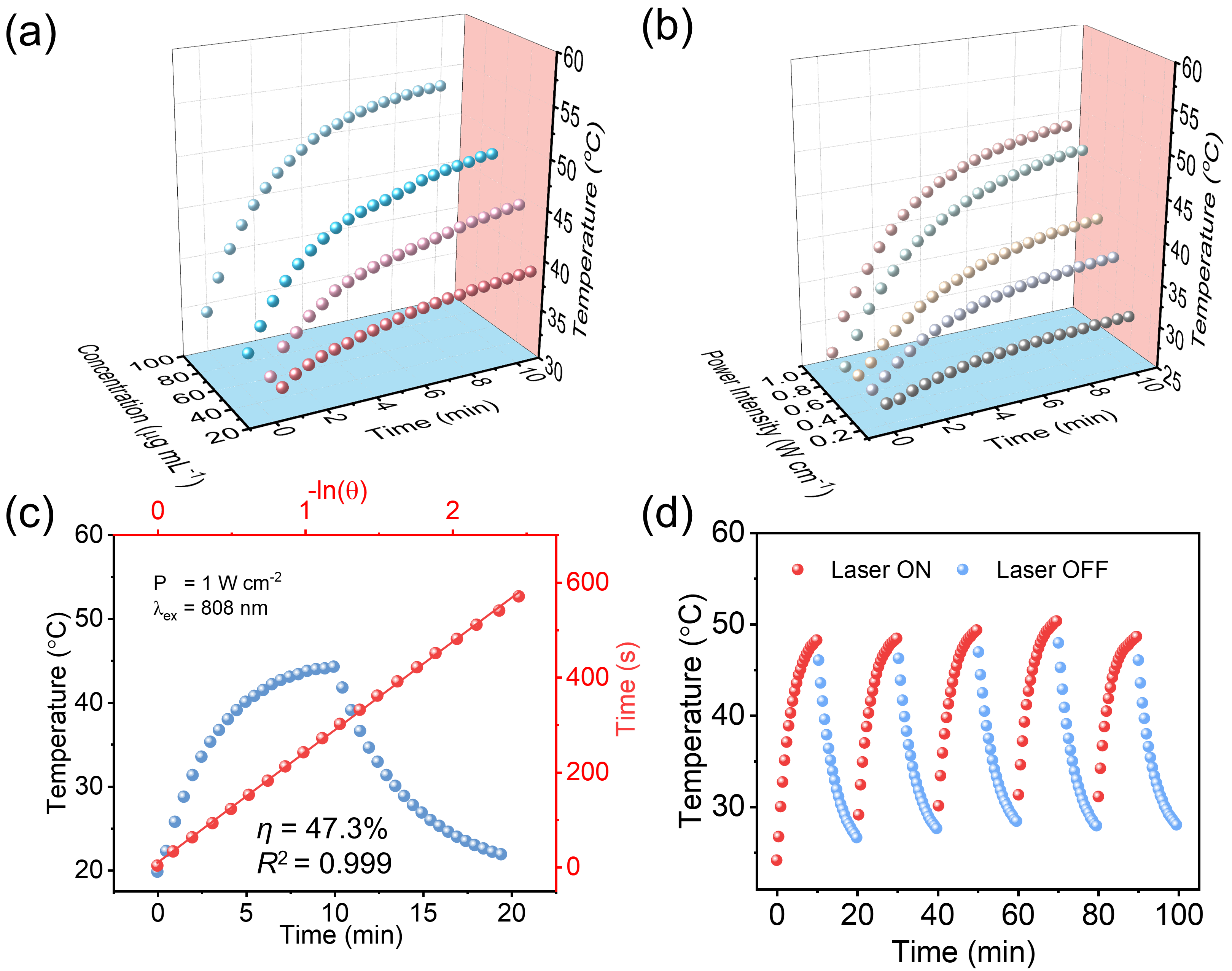
**

**Figure S10.** (a) Temperature changes of FBDFTPA NPs at different concentrations under irradiation (1.0 W cm^-2^). and (b) Temperature changes of FBDFTPA NPs (100 μg mL^-1^) at different laser power densities. (c) The photothermal conversion efficiency of FBDFDPA NPs under exposure of 808 nm laser (1.0 W cm^-2^).

**Table S3.** Currently reported NIR-II excitable small molecular dyes.

| **Entry** | **Chemical structure** | **molar extinction coefficients** | **PCE%** | **Reference** |
| --- | --- | --- | --- | --- |
| 1 |  | 14.4  L g^-1^ cm^-1^  (1060 nm) | 77 | Adv. Mater.  **2020**, *32*, e2001146 |
| 2 |  | 1.13 × 10^4^  M^-1^ cm^-1^  (1064 nm) | 47.6 | Angew. Chem. Int. Ed. **2023**, *62*, 202215372 |
| 3 |  | 9.14 × 10^4^  M^-1^ cm^-1^  (878 nm) | 31.6 | Mater. Horiz.  **2020**, *7*, 1379 |
| 4 |  | 1.24 × 10^5^  M^-1^ cm^-1^  (1024 nm) | 34.3 | Small **2021**, *17*, 2100398. |
| 5 |  | N.M. | 45.25 | Biomaterials science **2019**, *7*, 3165-3177. |
| 6 |  | 2.22 × 10^5^  M^-1^ cm^-1^  (1064 nm) | 34.3 | *Nat. Commun.* **2021**, *12*, 218. |
| 7 |  | 1.68 × 10^4^  M^-1^ cm^-1^  (1064 nm) | 54.1 | Angew. Chem. Int. Ed. 2024, 63, e202407307 |
| 8 |  | 17628  M^-1^cm^-1^  (1140 nm) | 49 | J. Am. Chem. Soc. **2023**, *145*, 26487 |
| 9 |  | 3.92 × 10^4^  M^-1^ cm^-1^  (1092 nm) | 90.98 | Angew. Chem. Int. Ed. **2024**, *63*, 202400372 |
| 10 |  | 2.93 × 10^4^  M^-1^ cm^-1^  (905 nm) | 62.5 | Angew. Chem. Int. Ed. **2025**, e202503718 |
| 11 |  | 7.66 × 10^3^  M^-1^ cm^-1^  (1064 nm) | 54.4 | This work |

**
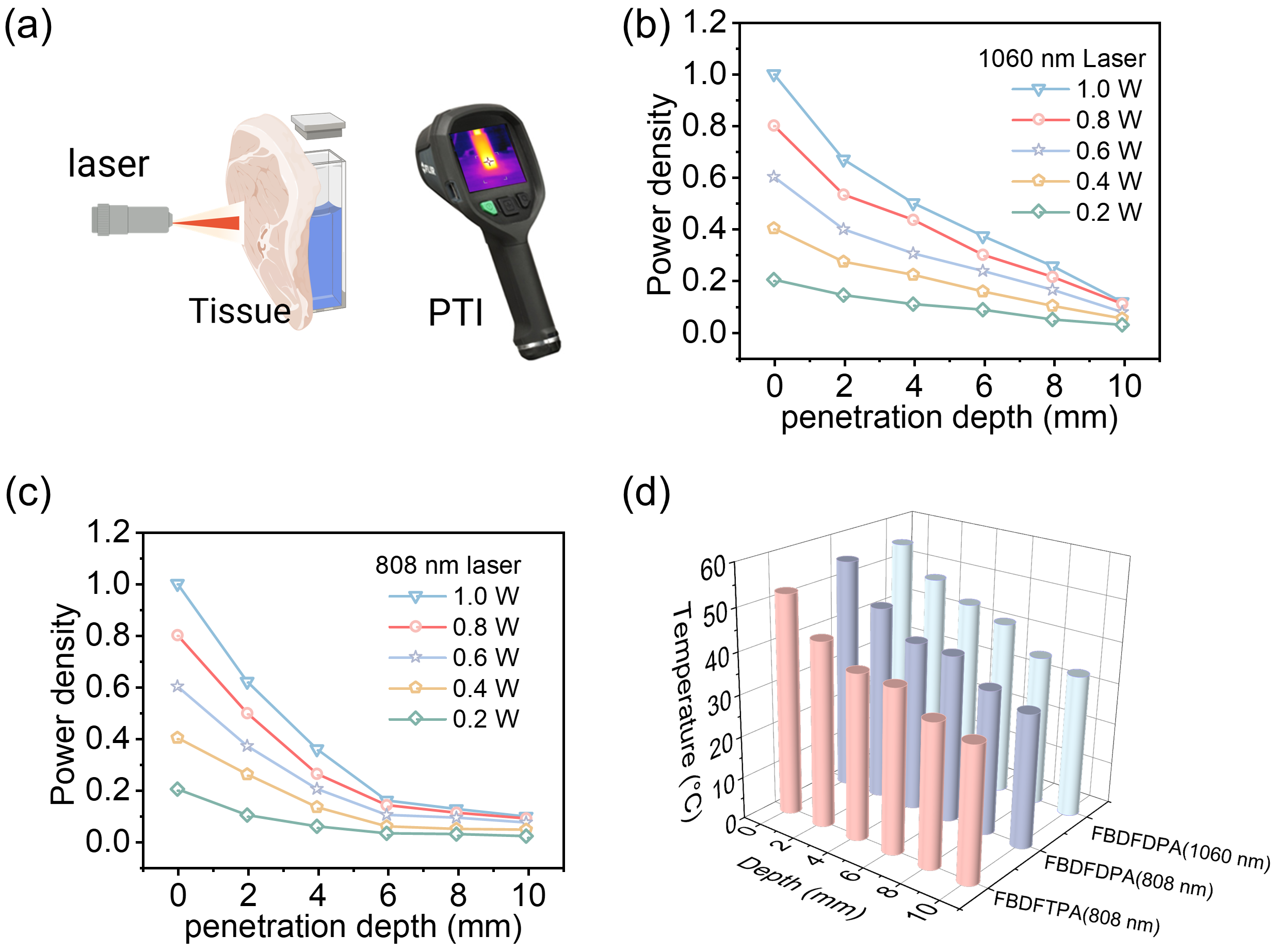
**

**Figure S11.** (a) Schematic diagram of different wavelength laser penetration evaluation.(b) Attenuation of the power density of chicken breast tissue with different thicknesses under 1060 nm laser irradiation. (c) Attenuation of the power density of chicken breast tissue with different thicknesses under 808 laser irradiation. (d) Comparison of temperature increment of FBDFDPA and FBDFTPA covering chicken breast tissue with different thicknesses under 808 and 1060 laser irradiation.

**
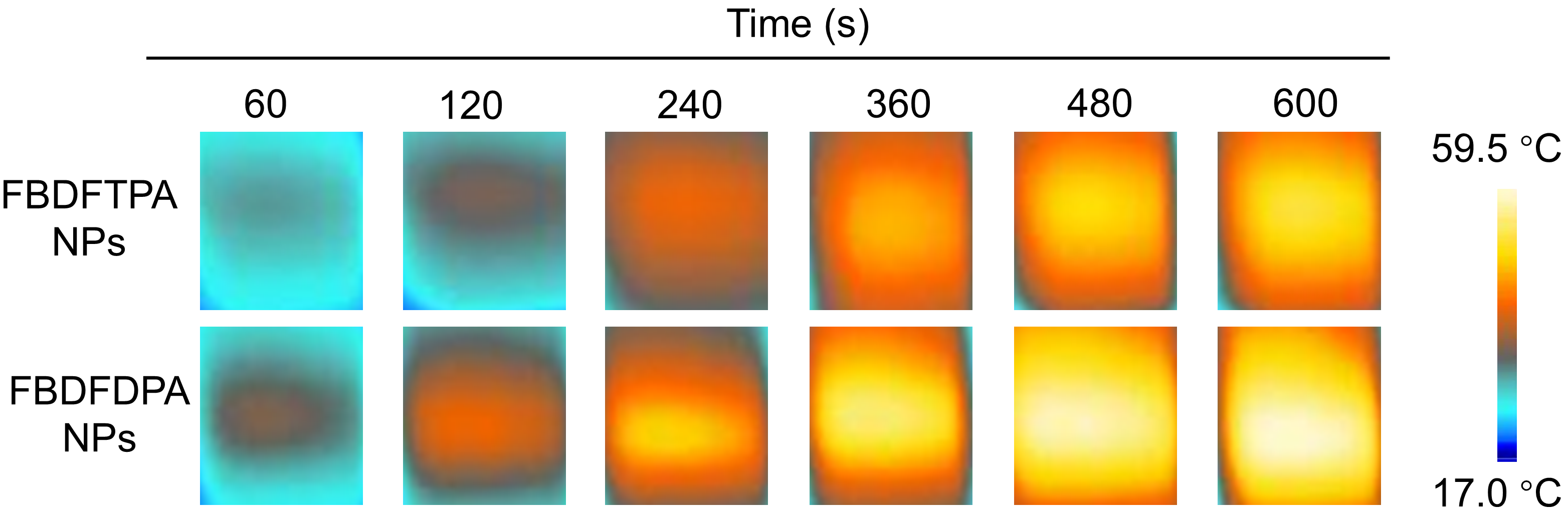
**

**Figure S12.** Photothermal images of FBDFTPA NPs under 808 nm laser irradiation, and FBDFDPA NPs under 1060 nm laser irradiation.

**
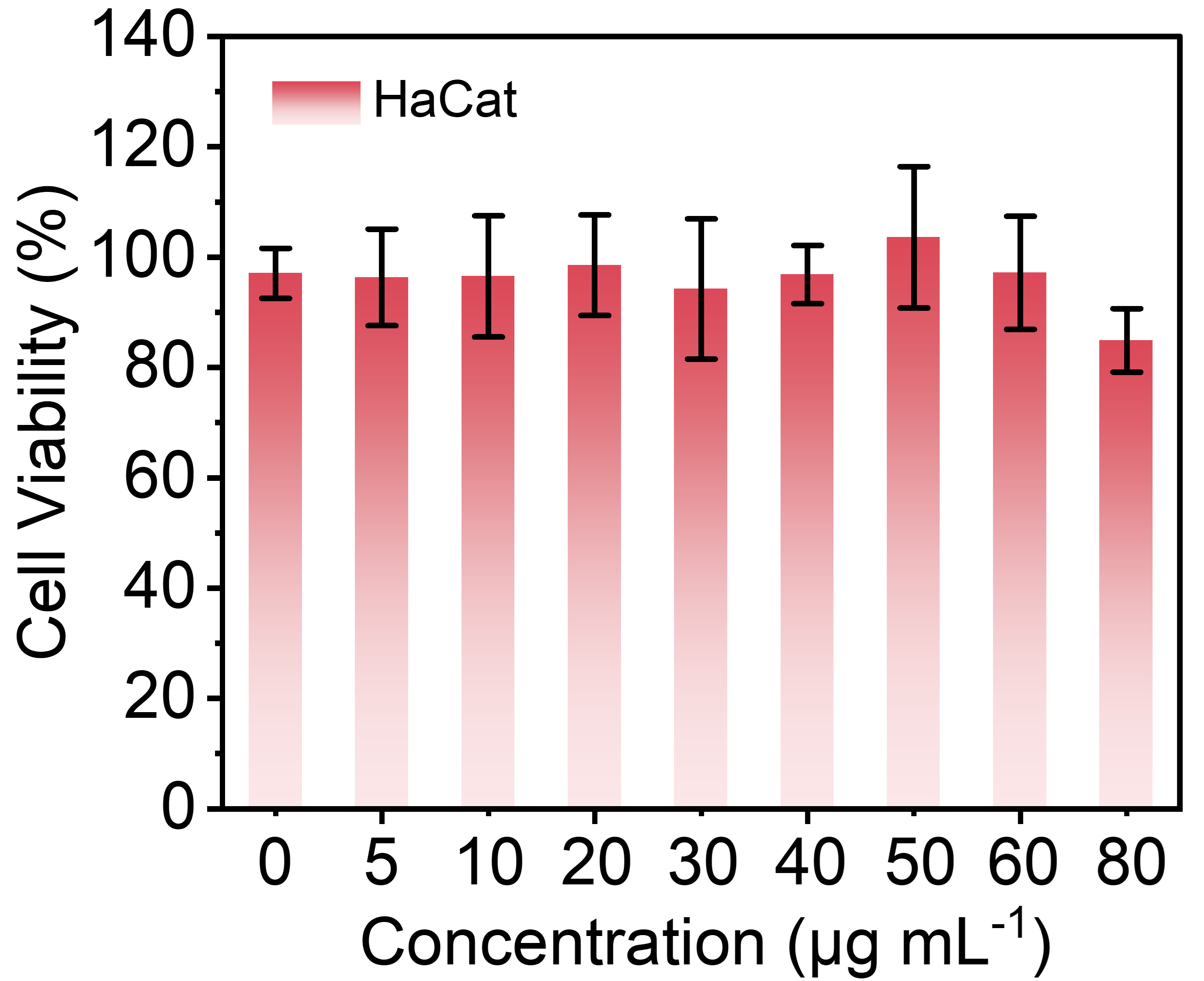
**

**Figure S13.** Viability of HaCat cells incubated with FBDFDPA NPs.

**
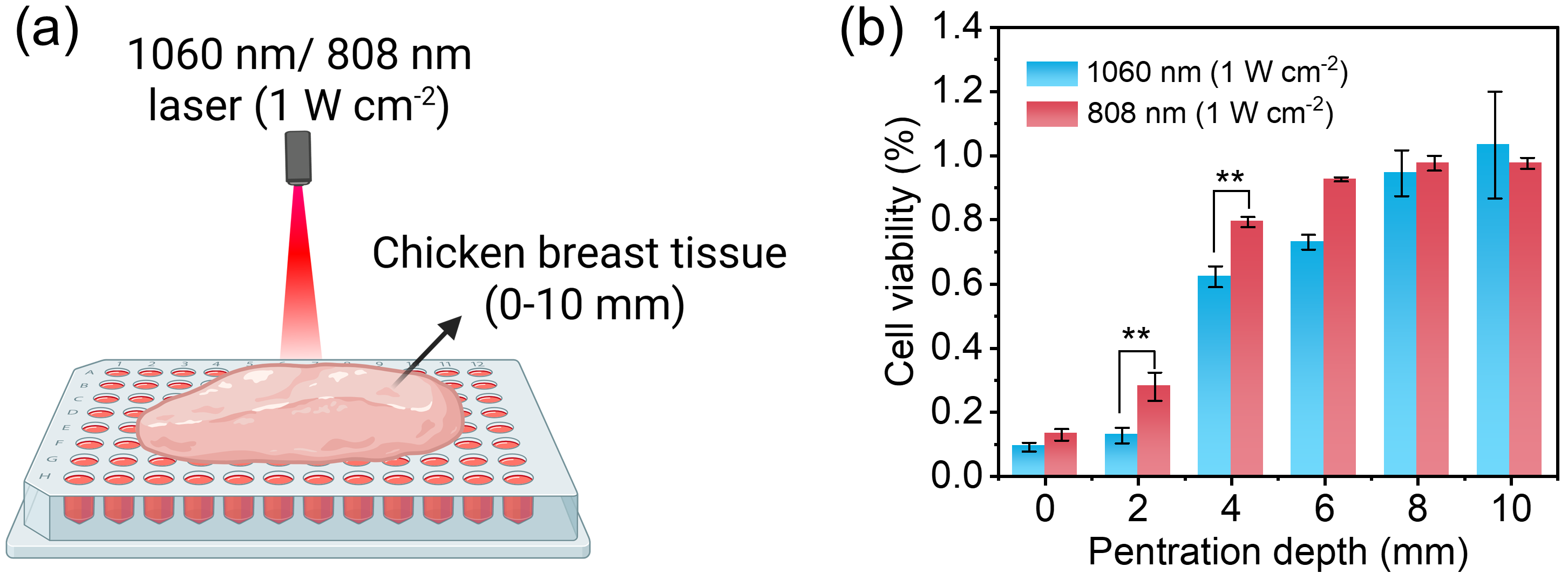
**

**Figure S14.** (a) Schematic diagram of the chicken breast penetration experiment. (b) Viability of 4T1 cells incubated with FBDFDPA NPs (100 µg mL^-1^) at different depths of chicken breast tissue under 1064 nm (1.0 W cm^-2^) and 808 nm (1.0 W cm^-2^) photoirradiation, **p < 0.01.

**
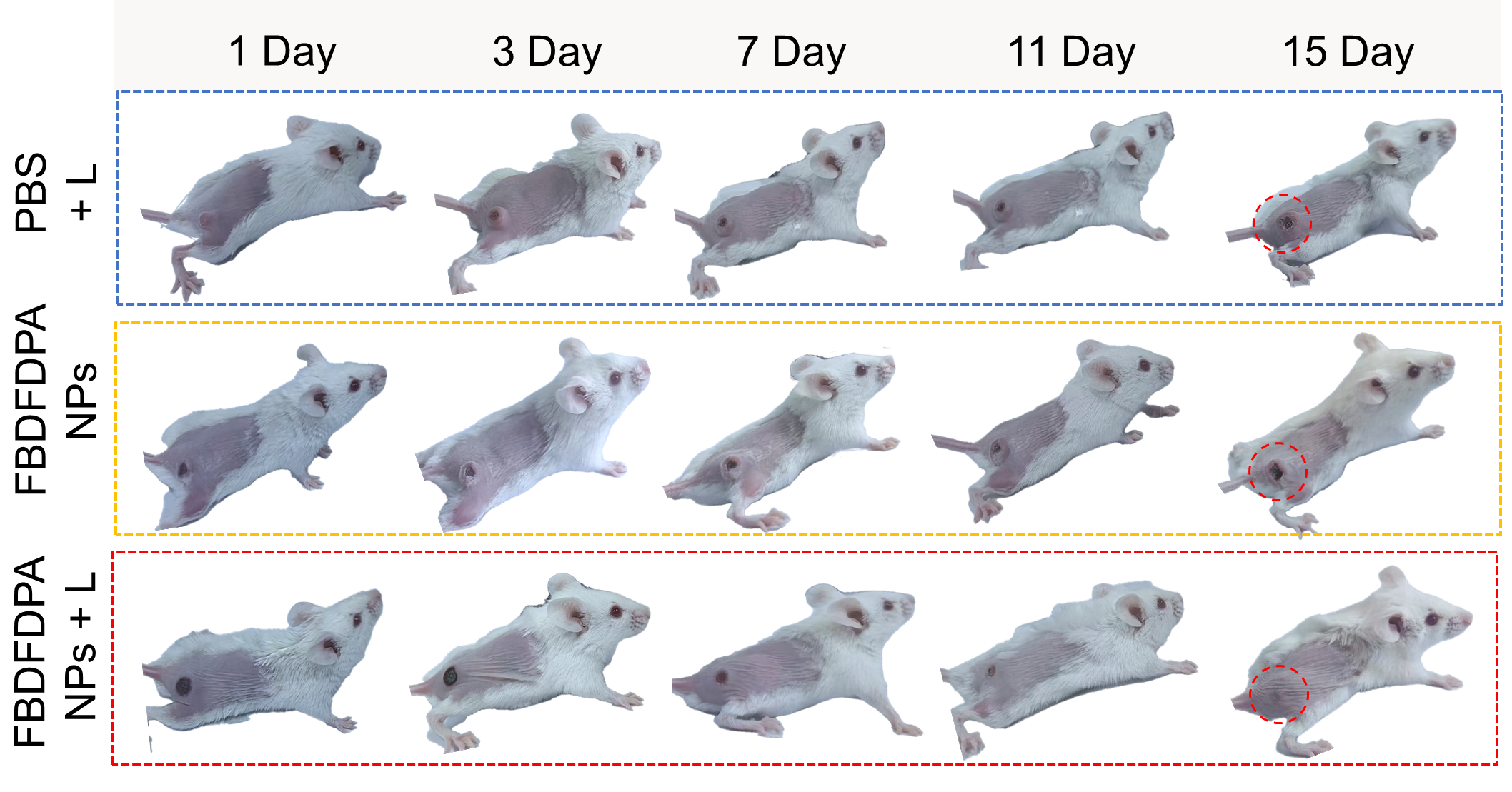
**

**Figure S15.** Digital photographs of mice during the 15-days treatment.

**
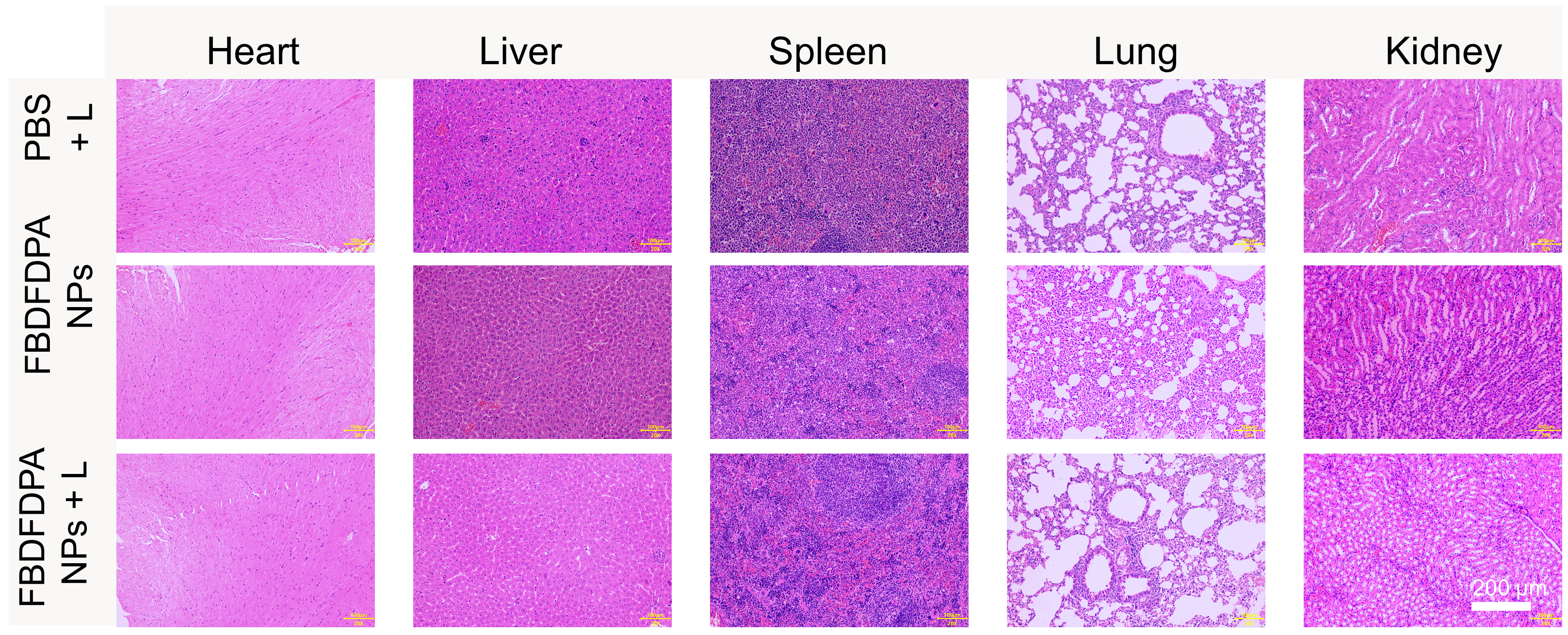
**

**Figure S16.** H&E staining images of major organs of mice.

**4. NMR and mass spectrometry data**

^1^H NMR spectrum of compound **1**.

^1^H NMR spectrum of compound **2**.

^1^H NMR spectrum of compound **3**.

**
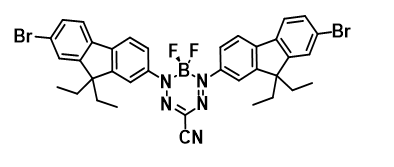
**

^1^H NMR spectrum of compound **4**.

^1^H NMR spectrum of FBDFTPA.

^13^C NMR spectrum of FBDFTPA.

MALDI-TOF Mass spectrum of FBDFTPA.

^1^H NMR spectrum of FBDFDPA.

^13^C NMR (100 MHz, CDCl_3_) spectrum of FBDFDPA.

MALDI-TOF Mass spectrum of FBDFDPA.
